# Trophic level drives the host microbiome of soil invertebrates at a continental scale

**DOI:** 10.1186/s40168-021-01144-4

**Published:** 2021-09-20

**Authors:** Dong Zhu, Manuel Delgado-Baquerizo, Jing Ding, Michael R. Gillings, Yong-Guan Zhu

**Affiliations:** 1 State Key Laboratory of Urban and Regional Ecology, Research Center for Eco-Environmental Sciences, Chinese Academy of Sciences, Beijing, 100085 China; 2 Departamento de Sistemas Físicos, Químicos y Naturales, Universidad Pablo de Olavide, 41013 Sevilla, Spain; 3 School of Environmental and Material Engineering, Yantai University, 30 Qingquan Road, Yantai, 264005 China; 4 Department of Biological Sciences, ARC Centre of Excellence in Synthetic Biology, Macquarie University, Sydney, NSW 2109 Australia; 5 Key Lab of Urban Environment and Health, Institute of Urban Environment, Chinese Academy of Sciences, 1799 Jimei Road, Xiamen, 361021 China

**Keywords:** Soil food web, Host microbiome, Unique microbial taxa, Biodiversity, Microbial dark matter, Continental-scale survey, Trophic dynamics, Deterministic process, Network analysis

## Abstract

**Background:**

Increasing our knowledge of soil biodiversity is fundamental to forecast changes in ecosystem functions under global change scenarios. All multicellular organisms are now known to be holobionts, containing large assemblages of microbial species. Soil fauna is now known to have thousands of species living within them. However, we know very little about the identity and function of host microbiome in contrasting soil faunal groups, across different terrestrial biomes, or at a large spatial scale. Here, we examined the microbiomes of multiple functionally important soil fauna in contrasting terrestrial ecosystems across China.

**Results:**

Different soil fauna had diverse and unique microbiomes, which were also distinct from those in surrounding soils. These unique microbiomes were maintained within taxa across diverse sampling sites and in contrasting terrestrial ecosystems. The microbiomes of nematodes, potworms, and earthworms were more difficult to predict using environmental data, compared to those of collembolans, oribatid mites, and predatory mites. Although stochastic processes were important, deterministic processes, such as host selection, also contributed to the assembly of unique microbiota in each taxon of soil fauna. Microbial biodiversity, unique microbial taxa, and microbial dark matter (defined as unidentified microbial taxa) all increased with trophic levels within the soil food web.

**Conclusions:**

Our findings demonstrate that soil animals are important as repositories of microbial biodiversity, and those at the top of the food web harbor more diverse and unique microbiomes. This hidden source of biodiversity is rarely considered in biodiversity and conservation debates and stresses the importance of preserving key soil invertebrates.

**Video abstract**

**Supplementary Information:**

The online version contains supplementary material available at 10.1186/s40168-021-01144-4.

## Background

Soil fauna are critically important for life on Earth [1–4]. They account for over one fifth of known animals and support key soil processes such as decomposition, nutrient cycling, and climatic influences by regulating the inputs and outputs of energy and matter within the soil food web [1, 5–7]. However, soil organisms are also highly vulnerable to global change. A number of recent studies indicated that they are facing a quiet extinction in terrestrial ecosystems across the globe, and especially so for the soil invertebrates at the top of the food web [8–11].

These animal extinctions might hide a deeper crisis, involving the potential loss of thousands of unique microbial species living within the microbiome of these soil fauna (Scenarios 1 and 2; Fig. S1), because recent studies have shown that diverse microbial communities inhabit soil fauna [12–15]. The impact of invertebrate extinctions would not be as severe for microorganisms if different soil invertebrates shared similar microbiomes (Scenario 3; Fig. S1) or if faunal microbiomes simply reflected the microbial communities in soil (Scenario 4; Fig. S1).

However, we lack even the most basic information on the identity and diversity of the microbiome for most soil invertebrates. Further, comprehensive studies evaluating the soil invertebrate microbiome across contrasting ecosystems and over large spatial scales have not been conducted. This hampers our ability to predict the consequences of soil faunal extinction on microbial biodiversity. We argue that investigating the biodiversity and identity of microbial taxa within the microbiome of functionally important and widely distributed soil invertebrates is fundamental to a complete understanding of food webs and biodiversity.

The soil fauna microbiome plays an important role in the health of its host and also affects ecosystem function [16–19]. Since the majority of the microbial taxa in soil fauna remain uncharacterized, this poses a major challenge to our understanding of their function. A network approach has been used to reveal the ecological contributions of this microbial dark matter. This identified a group of unknown taxa that clustered together, suggesting they performed essential ecological roles within the microbial community networks of the four extreme aquatic habitats examined [20]. We argue that soil animals also provide unique and distinct niches which can offer a habitat for low abundance environmental microbiota. While the functions of these microbiota are still poorly understood, they are likely to play critical roles in ecosystem function.

In addition, understanding the assembly of microbial communities is critical in microbial ecology [21–23]. Previous studies have showed that substantial microbial variation occurs among individuals in the soil fauna microbiome [12, 14, 15]. However, few studies consider how microbial communities are assembled in the wild soil fauna. A neutral community model was usually employed to assess microbial assembly [24, 25]. This model assumed (1) random sampling of OTUs from an equivalent microbial source pool, (2) equivalent immigration of OTUs among animals, and (3) equivalent microbial birth and death rates within animals. Collembolans, oribatid mites, and predatory mites live mainly in the pores of the soil, and nematodes, potworms, and earthworms depend on soil water for their activities. This indicates that environmental selection for microbiota within the collembolans, oribatid, and predatory mites may be different from nematodes, potworms, and earthworms due to differences in habitat. Differences in the environmental selection could affect random sampling and immigration of OTUs within soil fauna. Therefore, we hypothesized that the contribution of neutral processes to microbial assembly in collembolans, oribatid, and predatory mites would be different from that in nematodes, potworms, and earthworms.

Trophic levels of organisms are defined by diet [26–31], which in turn is known to influence the faunal microbiome [32–35]. Consequently, the complexity and identity of the microbiome of soil invertebrates could be strongly influenced by their hierarchical position within the food web. For example, predators in soil food webs might be generalists [28, 30, 36], which could ingest multiple low trophic organisms. This means that, apart from the environment, they could acquire microbiota from multiple low trophic organisms, and some microbes unique to different low trophic organisms might coexist within predators. Therefore, the predator might have a more diverse microbiome compared to a lower trophic organism. In addition, animals feeding on soil or plants might carry microbial communities that are similar to those in these environments [37]. Thus, identifying associations between trophic levels and microbial traits will be fundamental to understanding the real consequences of invertebrate extinctions (Scenarios 1 and 2; Fig. S1). Natural variations in stable isotope ratio of nitrogen (δ^15^N values) are commonly used to determine the trophic level of an organism in the food web. Because the information about what an animal ate at a certain time period is integrated in stable isotope values. The δ^15^N value of animals has a constant enrichment compared to their prey due to the preferential excretion of lighter isotope ^14^N. This stable isotope method is particularly useful for the soil ecosystem (cryptic system).

Here, our aims were to (1) examine the composition, diversity, and variation in the microbiome of each soil faunal group across different species, trophic levels, sampling sites, and landuse patterns; (2) elucidate ecological roles (e.g., within ecological networks) of microbial dark matter (defined as unidentified microbial taxa) in soil faunal microbiomes using network analysis; (3) reveal the assembly of soil faunal microbiome; and (4) explore the relationship between host microbiota and trophic levels within the soil food web, by employing bacterial 16S rRNA gene sequencing and nitrogen stable isotopes. To address these aims, we analyzed the microbiomes of six groups of invertebrate soil fauna from contrasting ecosystems across China. Both farmland and forested lands were covered. The six groups included some of the most dominant and functionally important soil invertebrates on Earth (see Additional file 3): collembolans (springtails), nematodes, potworms, earthworms, oribatid mites, and predatory mites [3, 4, 30]. Each of these have very different diets and positions within the soil food web. The study spanned a north/south gradient that covered most climatic zones in China (Fig. S2) and consequently was a cross-biome investigation.

## Methods

### Collection sites and sampling procedure

Sampling was performed at six sites during October and November 2017, at locations between 24.9° and 41.7° N and between 102.95° and 123.72° E, covering most climatic zones from north to south in China (Fig. S2). Two contrasting terrestrial ecosystems (farmland and natural forest) adjacent to each other were sampled at each site. At each location, five replicate soil and faunal samples were collected from the top 0–6 cm surface layer of soil. For animal samples, a block of soil (length 50 cm, width 35 cm, and depth 6 cm) was taken, stored at 4^o^C, and brought back to the laboratory within 24 h. Since soil animals are mobile, they can “collect” microorganisms from the possible range of soil animal activity (16 m^2^). Consequently, we collected soil samples, which also covered basically this range. Soil samples were preserved in dry ice and taken back to the lab within 24 h.

### Isolation of soil fauna and DNA extraction

After samples were transported to the lab, extraction of all soil animals was conducted within 8 h to minimize variation in microbial communities. Earthworms were collected from soil by hand, using gloves. Earthworms were successfully collected at five sites (expect for Shenyang), and fifty earthworm community samples were obtained in all. Due to the sensitivity of soil collembolans and mites to heat, a controlled-temperature gradient extractor was used to isolate them from soil [13], which is an improvement on the Berlese dry extraction. To minimize potential shifts in the host microbiome, the temperature of the receiver (containing 2-cm depth of absolute alcohol) was set at 4°C, where collembolans and mites were collected. The extraction procedure was conducted over 6 h. A modified Baermann wet funnel was used to extract potworms and nematodes, also across a 6-h period [12].

Communities of collembolans, nematodes, potworms, earthworms, and mites were successfully collected, with a large number of individuals obtained (7200 collembolans, 18000 nematodes, 3000 potworms, 2000 earthworms, 8000 oribatid mites, and 4000 predatory mites). In our study, the predatory mite that feeds on other animals included mesostigmatids and predatory prostigmatids. Each faunal category contained many species. Considering the objective of our study and the biomass of some species, we only selected some dominant species of soil fauna at each site to further analyze their microbiomes and to analyze ^15^N isotope signatures. Since the collembolan and mite commonly occupied multiple trophic levels, we analyzed more than one species at each site in our study. Although the nematodes also occupied different trophic levels in the soil food web, the biomass of many nematode species was too low to meet the analysis requirements. Thus, we only selected one dominant species per site from obtained nematode communities for downstream analysis. The dominant soil fauna was identified to the species level using morphological features and were stored in the absolute alcohol until DNA extraction. DNA barcoding was then used to confirm species identity as a supplement to morphological identification. Soil faunal DNA was amplified from each sample using universal primers (see Table S1), sequenced at the Beijing Genomics Service, edited using Genious, and then used to interrogate the NCBI database using BLASTn. Sequence matches greater than 97% were taken as species identities. Since the diversity of dominant species of soil fauna was different at each site, we obtained different number of soil animal samples. The list of sampling information and soil animal species used in our study were given in the Additional file 2. The description of how our soil fauna samples correspond to each of the five fauna samples collected per site was also provided in the Additional file 2, and the numbers of soil fauna samples from each extraction varied per collected site. In total, 238 collembolan samples (about 3600 individuals), 60 nematode samples (about 6000 individuals), 62 potworm samples (about 1500 individuals), 146 oribatid mite samples (about 4000 individuals), 122 predatory mite samples (about 2000 individuals), 50 earthworm samples (about 1000 individuals), and 60 soil samples were analyzed, and microbial communities and ^15^N isotope signatures were also determined. Each soil fauna sample was analyzed at the species level, and five soil samples were analyzed for each site.

After species identification based on morphology and DNA barcoding, individuals of each selected species were pooled to isolate DNA. Soil fauna (nematodes 30 individuals, collembola 5 individuals, potworms 3 individuals, oribatid mites 5 individuals, predatory mites 5 individuals, and earthworms 3 individuals per sample) were surface sterilized by washing three times in 0.5% sodium hypochlorite, and rinsed five times with sterile ultrapure water before DNA extraction [38]. The surface-sterilized animals were transferred into a sterile centrifuge tube and were homogenized using a micro-electric tissue homogenizer. Finally, a DNeasy Blood and Tissue Kit (QIAGEN, Germany) was used to isolate DNA of the soil fauna microbiome based on the manufacturer’s instructions. At least 30 ng of DNA was extracted from each soil faunal sample, and the DNA concentration of different animals was unified to 1 ng μL^-1^ before PCR. We obtained soil DNA using a FastDNA® Spin Kit for Soil (MP Biomedical, USA), following the manufacturer’s instructions. DNA was stored at −20°C.

### 16S rRNA gene amplification, library preparation, MiSeq sequencing, and bioinformatics analysis

The 515f/806r primer set was used to amplify the V4 hypervariable region of the 16S rRNA gene, in triplicate, from the extracted DNA. Primers were barcoded to distinguish samples. PCR was conducted in 50 μL volumes, including 1 μL each of primers (5 μM), 1 μL animal DNA template (1 ng), 22 μL sterile ultrapure water, and 25 μL ExTaq (2×). Reaction conditions for DNA amplification and purification of the product were based on previously published methods [38, 39]. We determined the concentration of purified PCR products using a Qubit™ 3.0 fluorometer (Invitrogen, USA). Equal concentrations of PCR products (300 ng) were used in library construction, and each library contained 30 samples. Finally, we used the Illumina MiSeq platform to sequence the PCR libraries at the Meiji Biological Medicine Company, Shanghai, China.

Bacterial 16S rRNA gene data were processed using Quantitative Insights Into Microbial Ecology (Qiime version 1.9.1) according to the online instructions [40]. In short, high-quality sequences were obtained by removing labelled barcodes, low-quality reads, and ambiguous nucleotides. We combined the obtained sequences to operational taxonomic units (OTUs) based on 97% sequence similarity using open-reference OTU picking [41]. Singletons were removed, and PyNAST used to align representative sequences to each OTU [42]. The phylogenetic tree of representative sequences was constructed by using the Fast tree algorithm. The SILVA (v138) SSU reference database was selected to assign the taxonomy of each OTU using the RDP Classifier 2.2 [43]. OTUs with <100 reads across all samples (including soils and animals) were removed to minimize potential errors in sequencing [39]. Finally, we rarefied samples to a depth of 13,551, which discarded eight samples and produced a total of 730 samples used for downstream analyses. Basic characterizations of microbial community in the soil food web were summarized in the Supplementary Text (Figs. S3 and S4).

### Measurement of nitrogen stable isotope

We used a Delta V Advantage isotope ratio mass spectrometer (Thermo Finnigan, USA) to detect ^15^N isotope signatures of soil faunal whole bodies (at the species level) and plant litter [29]. For soil faunal samples, the number of each selected species (nematodes 50 individuals, collembola 10 individuals, potworms 8 individuals, oribatid mites 10 individuals, predatory mites 5 individuals, and earthworms 5 individuals) was different. For soil fauna with large biomass, we ground them into powder and took part of them to determine ^15^N isotope signatures. Plant litter was collected from each sampling site to determine the ^15^N isotope signature of primary producers. The urea was used as an internal reference for the quality control and measured once per 10 samples. The measured precision was < 0.10‰. The ^15^N isotope natural abundance was expressed using δX notation which indicates the deviation from the reference in parts per thousand (‰). The calculation of δX value was used the following formula [29]: δN = ((*R*_sample_ – *R*_reference_)/*R*_reference_) × 1000. The *R*_sample_ indicates ^15^N/^14^N of the sample, and *R*_reference_ indicates ^15^N/^14^N of the reference. In this study, we used atmospheric N_2_ (air) as the reference value of ^15^N. Natural ^15^N fractionation of animal body tissue (δ ^15^N value) is usually used to represent the trophic level of animal in the ecosystem. According to previous studies, we set 3.4 (the difference of δN value between samples) as the limit of different trophic level [29, 44]. In other words, we defined the difference of δN value between each tier of trophic level as 3.4. To ensure the comparability of δ ^15^N values between different sites, we determined δ ^15^N value of litter at each site and then obtained the adjusted δ ^15^N value by the measured δ ^15^N value of animal minus δ ^15^N value of litter. The adjusted δ ^15^N value was used in the downstream analysis.

### Statistical analysis

Alpha diversity (observed species and Shannon index) of bacterial communities were calculated using alpha_diversity.py in Qiime with version 1.9.1 [40], and the data were presented with mean ± standard error (SE). The adipart function of vegan 2.5–6 package of *R* [45] was used to perform additive diversity partitioning [46], which could reveal the relative contribution of individual, soil faunal group, sampling site, and landuse to soil faunal microbial diversity. Principal coordinates analysis (PCoA) was selected to reveal the difference in bacterial communities between different samples using the weighted unifrac distance based on the relative abundance of bacterial OTUs and the unweighted unifrac distance based on the presence/absence of bacterial OTUs, which was conducted in Qiime with version 1.9.1 using beta_diversity_through_ plots.py [40]. Enterotyping of each soil faunal group was clustered using the Jensen–Shannon distance and partitioning around medoids method based on the relative abundance of bacterial genera, which reveals differences in microbial community structure among soil faunal individuals not directly associated with environmental factors and faunal species [47–50]. We used the PERMANOVA (Adonis test) to assess significant differences in bacterial communities between different groups of samples. Analysis of variance (ANOVA) with the linear mixed model was used to compare differences in alpha diversity, δ ^15^N values, and community dissimilarity between different groups of samples by the IBM SPSS with version 22. If the data did not fall into a normal distribution, we used the generalized linear model with Poisson distribution to compare differences between samples. Venny 2.1 on-line was used to obtain OTUs shared between different animal groups and all soil samples.

We used the neutral community model of Sloan et al. [25] to assess the contribution of neutral processes to each soil faunal group microbial assembly, which was calculated via the R code of Burns et al. [24]. The phylogenetic bin-based null model analysis (iCAMP) was selected to quantify the relative importance of different ecological processes in the soil food web microbial assembly [51]. Violin plots showed unique bacterial taxa in different soil fauna microbiomes by filtering all collected soil microbial species from the faunal microbiome using the ggplot2 package of R. The composition of the 50 most abundant unique bacterial taxa in different soil fauna microbiomes was presented using the pheatmap package of R. FEAST (fast expectation-maximization for microbial source tracking) was used to estimate the contribution of source to the sink (predatory mite) in each sampling site [52]. The functional profile of the soil food web microbiome was predicted using Tax4Fun2 [53]. Linear fitting between trophic level (δ ^15^N value) and microbial diversity and predicted microbial functional diversity (Shannon index) was performed in Origin 2017. We employed the most abundant 258 unique bacterial taxa (maximal read abundance > 5%) to build a phylogenetic tree using Qiime and iTOL and used representative sequences of 258 unique bacterial taxa to compare with sequences that exist in the database via BLASTn to obtain the information of genome match at > 97% 16S rRNA sequence similarity level. We analyzed the change in dominant unique bacterial taxa (maximal relative read abundance > 1% and those found in more than 50% of soil fauna samples) along with the alteration of soil fauna trophic level using the pheatmap package. We also identified 26 dominant bacterial taxa based on a relative read abundance > 0.1% and being found in more than 80% of soil fauna samples, mainly focusing on those bacterial taxa that were high abundance and reasonably ubiquitous in multiple fauna [54]. We used random forest modeling to determine the accuracy with which soil fauna microbiomes could be assigned to their own type or trophic level according to 57 bacterial taxa (maximal relative read abundance > 0.3% and those found in more than 70% of soil fauna samples), which was performed in the randomForest 4.6–14 package with 1000 trees per model [55]. The confusion matrix of random forest modeling was presented with a heatmap in the pheatmap package of R. The Unknown taxa (including “unknown,” “unassigned,” “ambiguous taxa,” “uncultured,” “uncultured bacterium,” or “NA”) network was constructed using the package SpiecEasi (version 1.0.7) of R, and significant difference of betweenness, degree, and closeness between different networks was analyzed using the Wilcox test [20]. According to the measured δ ^15^N value, five trophic levels were determined in all soil fauna samples. We set significant difference (*P*) at 0.05 level. Other graphics were all produced in Origin 2017.

## Results

### The variation of microbial community among soil faunal microbiomes

**Fig. 1:**
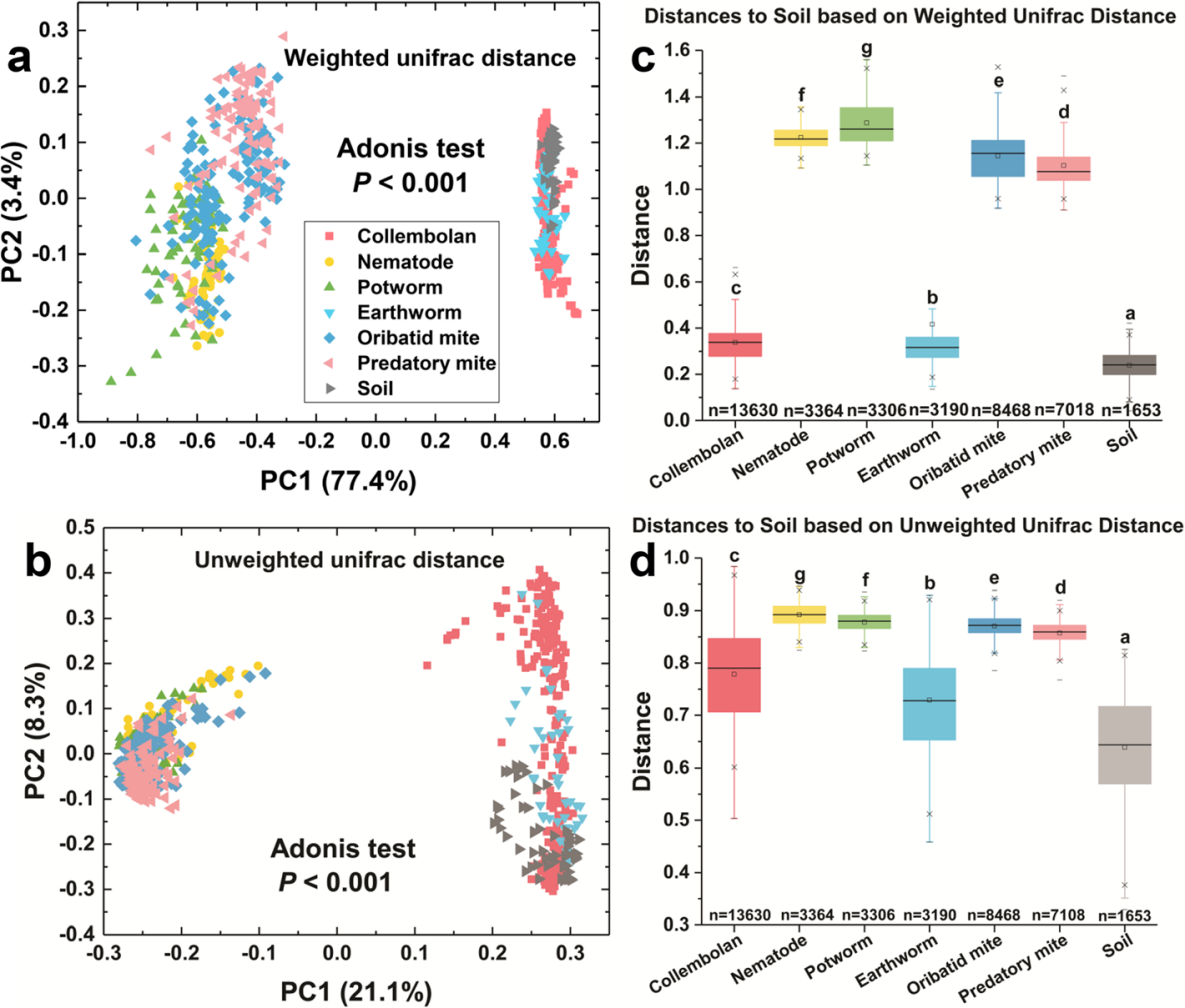
Principal coordinates analysis (PCoA) revealing the distribution of soil and fauna bacterial communities using the weighted unifrac distance based on the relative abundance of bacterial OTUs (**a**) and the unweighted unifrac distance based on the presence/absence of bacterial OTUs (**b**). Different shapes and colors represented different types of samples. The variation explained by the PCoA axes is listed in parentheses. Significant analysis of variance used distance matrices (PERMANOVA) via Adonis test (999 permutations). Boxplots presenting the distance of bacterial communities between each soil fauna group and soil samples (**c** weighted unifrac and **d** unweighted unifrac), which reflecting the similarity of bacterial community between treatments. Significance of results was evaluated using pairwise PERMANOVA and labeled using different letters (significant level *P* < 0.05). The “*n*” indicated the number of distance between each soil fauna group and soil. Centre line, median; box limits, first and third quartiles; whiskers, 1.5 × interquartile range

The total microbial diversity was partitioned to reveal the contribution of each sampling level to the diversity. The majority of soil faunal microbial diversity was partitioned at the individual level (66.7%; Table 1). The contribution of soil fauna group (10.6%), sampling site (8.2%), trophic level (5.1%), and landuse (1.2%) to microbial diversity were all significant (*P* < 0.01; Table 1). Some clustering of samples by types of samples was observed in the principal coordinate analysis, more distinctly for the weighted unifrac distance (*F*_6,723_ = 497.6, *P* < 0.001; Fig. 1a) than the unweighted unifrac distance (*F*_6,723_ = 43.67, *P* < 0.001; Fig. 1b). Along the PC1 axis, soil, earthworm, and collembolan samples were clustered and separated distinctly from other soil faunal samples (Adonis, *P* < 0.001; Fig. 1a, b). This was consistent at each sampling site (Figs. S5 and S6). We also found that collembolan and earthworm microbiomes shared more OTUs with soil than did other soil fauna (Fig. S7). The distance of microbial community between soil fauna and soil samples was significantly greater than that between soil samples (*P* < 0.001; Fig. 1c, d). Host group, species, and trophic level all had a strong and significant impact on the variation of soil faunal microbiomes (*P* < 0.001; Table S2). A small but significant effect on variation among microbiotas was observed from the sampling site, only explaining < 2% of the variation (*P* < 0.001; Table S2).

**Table 1:** Hierarchical partitioning of diversity within and among soil fauna microbiomes

Alternative hierarchy schemes	Diversity level	Description	Shannon		
			Index	%	*P*
1. Unstructured	Gamma	Total diversity	7.72	100	-
2. Individuals and soil fauna groups	Alpha	Average alpha diversity of an individual	5.15	66.7	<0.01
	Beta 1	Among individuals of a soil fauna group	1.75	22.7	<0.01
	Beta 2	Among soil fauna groups	0.82	10.6	<0.01
3. Individuals and sampling sites	Alpha	Average alpha diversity of an individual	5.15	66.7	<0.01
	Beta 1	Among individuals of a sampling site	1.94	25.1	<0.01
	Beta 2	Among sampling sites	0.63	8.2	<0.01
4. Individuals and land use patterns	Alpha	Average alpha diversity of an individual	5.15	66.7	<0.01
	Beta 1	Among individuals of a land use pattern	2.48	32.1	<0.01
	Beta 2	Among land use patterns	0.09	1.2	<0.01
5. Individuals and trophic levels	Alpha	Average alpha diversity of an individual	5.15	66.7	<0.01
	Beta 1	Among individuals of a trophic level	2.18	28.2	<0.01
	Beta 2	Among trophic levels	0.39	5.1	<0.01
6. Individuals, sampling sites, and land use patterns	Alpha	Average alpha diversity of an individual	5.15	66.7	<0.01
	Beta 1	Among individuals of a sampling site at a land use pattern	1.95	25.2	<0.01
	Beta 2	Among individuals of a land use pattern	0.53	6.9	<0.01
	Beta 3	Among land use patterns	0.09	1.2	<0.01

For each soil faunal group, the principal coordinate analysis revealed many samples clustered by sampling site (Figs. S8 and S9). The Adonis analysis further indicated that sampling site had a significant and strong effect on microbial variation (*P* < 0.001; Fig. S10). We also found that host species and sampling site all had a strong selection on the microbiome of each soil faunal group (Fig. S10), and the order of strength of the host selection was collembolan (41.84%) > potworm (31.50%) > nematode (28.09%) > oribatid mite (27.07%) > earthworm (22.93%) > predatory mite (15.56%).

We further identified two enterotype clusters in collembolan (Ca and Cw), nematode (Nc and Ng), earthworm (Ebu and Eba), and predatory mite (Pa and Pr) microbiomes, and microbiomes of potworm (Pf, Ps, and Pa) and oribatid mite (Os, Oa, and Og) were sorted into three robust enterotypes (Fig. S11), respectively. Each soil faunal group contained the enterotype, which overrepresented the bacterial genus *Acinetobacter*. For the predatory mite, most samples (92.8%) were identified into Enterotype Pa, including abundant *Acinetobacter*. Enterotypes were overrepresented in the sampling site. For example, all Enterotype Pr (*Rickettsiella*) were found in the microbiome of predatory mites collected from Kunming.

### Microbial dark matter in the soil food web

Class-level and genus-level network analysis all revealed that unknown taxa presented distinctive clusters in the soil food web microbial network (Fig. S12a and S12b). More frequently shared edges were found between unknown taxa than between unknown and classified OTUs in the soil food web microbial network (Fig. S12c). However, for each soil faunal group, unknown taxa were all intermixed with known OTUs at the genus level (Fig. S13). Removal of the unknown taxa significantly reduced centrality scores of betweenness and degree in the soil food web network metric across all taxonomic levels compared to the Original and Bootstrap networks (*P* < 0.01; Fig. S14 and Table S3). A significant increase in the closeness was observed in the soil food web without unknown taxa network at the genus level but reduction at the other taxonomic levels (*P* < 0.001; Fig. S14 and Table S3). For each soil faunal group, without unknown taxa network metrics all had higher centrality values of closeness and lower betweenness and degree than those in the original network across multiple taxonomic levels (*P* < 0.05; Table S3). At the genus level, many top hub scores were unknown taxa for the soil food web microbial network, and over half of the nodes within the top 50 hub scores were unknown OTUs for nematode, potworm, and predatory mite networks (Table S3).

### Microbial community assembly in the soil food web

**Fig. 2:**
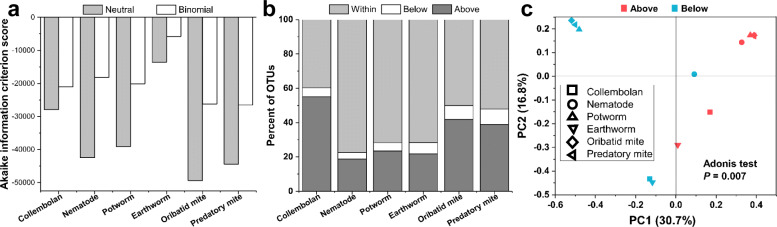
Characteristics of neutral models for each soil fauna group. **a** The comparison of Akaike information criterion (AIC) scores between a neutral model fit and fit of a binomial model. **b** The percent of OTUs from each soil fauna group that fall within, below, and above neutral model prediction. **c** Principal coordinates analysis presenting the distribution of bacterial communities of partitions above and below neutral model predictions, based on the presence/absence of OTUs using the Jaccard index

**Fig. 3:**
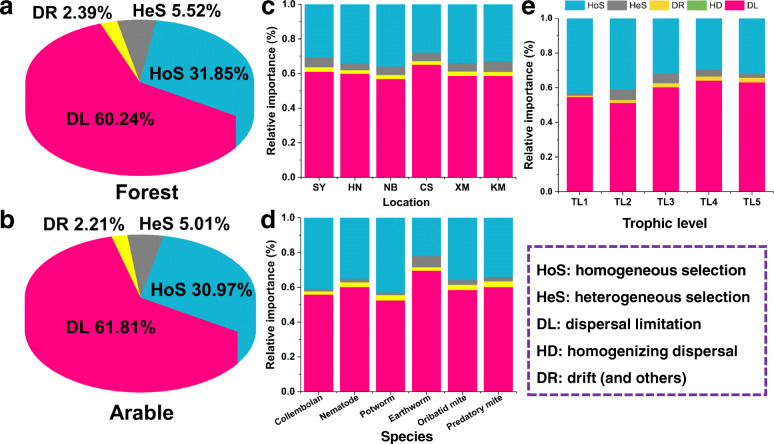
Relative importance of different ecological processes in response to landuse (**a**, **b**), sampling site (**c**), soil faunal group (**d**), and trophic level (**e**), which was estimated by phylogenetic bin-based null model analysis (iCAMP)

The Sloan neutral community model was employed to evaluate the effect of neutral processes on the assembly of each soil faunal group microbiome. Lower Akaike information criterion (AIC) scores were observed in the neutral model compared to the binomial model for all soil faunal groups (Fig. 2a). The distribution of bacterial taxa could largely be explained by neutral processes in nematode (77.4%), potworm (71.7%), and earthworm (71.6%) microbiomes in comparison to those in collembolan (39.7%), oribatid mite (50.1%), and predatory mite (52.1%) (Fig. 2b). The percent of OTUs below the neutral model prediction was much lower than that above in all soil faunal groups. The principal coordinates analysis revealed that bacterial communities of above neutral model predictions were distinctly separated from those in the below (*P* = 0.007; Fig. 2c). The order of the Nm-value for different soil faunal groups was collembolan (4026) > predatory mite (2103) > earthworm (1802) > oribatid mite (1369) > potworm (999) > nematode (667) (Fig. S15). We further used phylogenetic bin-based null model analysis to quantitatively assess microbial assembly processes, which showed homogeneous selection (31.41%) and dispersal limitation (61.02%) played dominant roles in soil food web microbial assembly (Fig.3). The relative importance of stochastic processes (including dispersal limitation, homogenizing dispersal, and drift) was 62.63% under forest and 64.02% under farmland (Fig. 3a and b). The relative importance of stochastic processes was the highest in the sampling site Changsha (67.29%; Fig. 3c) and earthworms (71.63%; Fig. 3d), respectively. Overall, an increase in the stochastic processes was associated with an increase in the trophic level (Fig. 3e).

### Change of soil faunal microbial traits with increasing trophic level

The position of soil fauna within the food web was identified using nitrogen isotopes, since higher trophic levels are enriched for δ ^15^N. The natural δ ^15^N value of soil faunal body tissues showed a significant difference between different animals (Fig. S16; *P* < 0.001), ranging from −0.25 to 16.79 (representing approximately five trophic levels). Noteworthily, collembolans covered a wide range of trophic levels (~5), and predatory mites had the highest δ ^15^N value (mean 11.1) compared to other soil fauna (*P* < 0.001; Fig. S16), suggesting that predatory mites occupied the highest trophic level in the selected soil food web. The FEAST analysis showed that the sources of predatory mite microbiome exhibited differences in different sampling sites, and collembolan, nematode, potworm, earthworm, and oribatid mite all had an important contribution to the predatory mite microbiome, especially the oribatid mites (Fig. S17). The Venn diagram also showed that most OTUs (98%) were shared between different trophic levels of soil faunal microbiomes (Fig. S18). The heatmap further revealed that individual taxa of some dominant bacteria (e.g. the dominant bacteria in the genera *Acinetobacter* and *Rhodoplanes*) in the soil faunal microbiomes were consistently associated with trophic level (*P* < 0.001; Fig. S19). Therefore, we employed random forest modeling to examine the predictive ability of the soil invertebrate microbiome for the type (predictive accuracy: 91.9%) and trophic level (predictive accuracy 66.7%), based on 58 dominant bacterial taxa (Fig. S20).

**Fig. 4:**
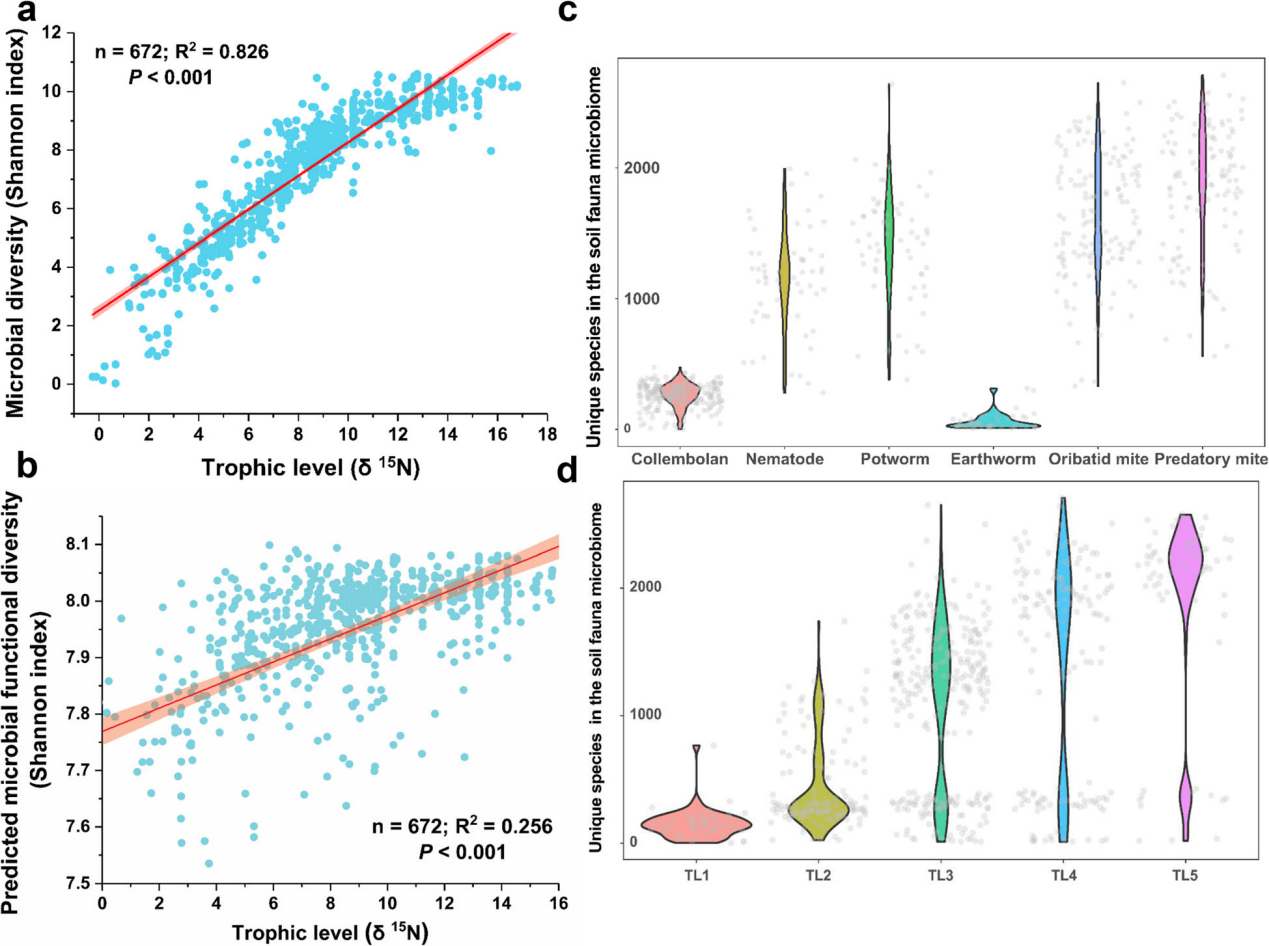
Linear regression revealed the relationships between soil fauna trophic level (δ ^15^N value) and microbial diversity (**a**) and predicted microbial functional diversity (**b**). Violin plots showed unique bacterial taxa in different soil fauna microbiomes by filtering all species found in soil microbiome from the faunal microbiome in different types of animals (**c**) and in different trophic level of animals (**d**). By “unique,” we mean species which were present in the microbiome of a given soil invertebrate, but were not detected in any soil samples. The “TL” indicated the trophic level of soil fauna based on natural ^15^N fractionation of animal body tissue

**Fig. 5:**
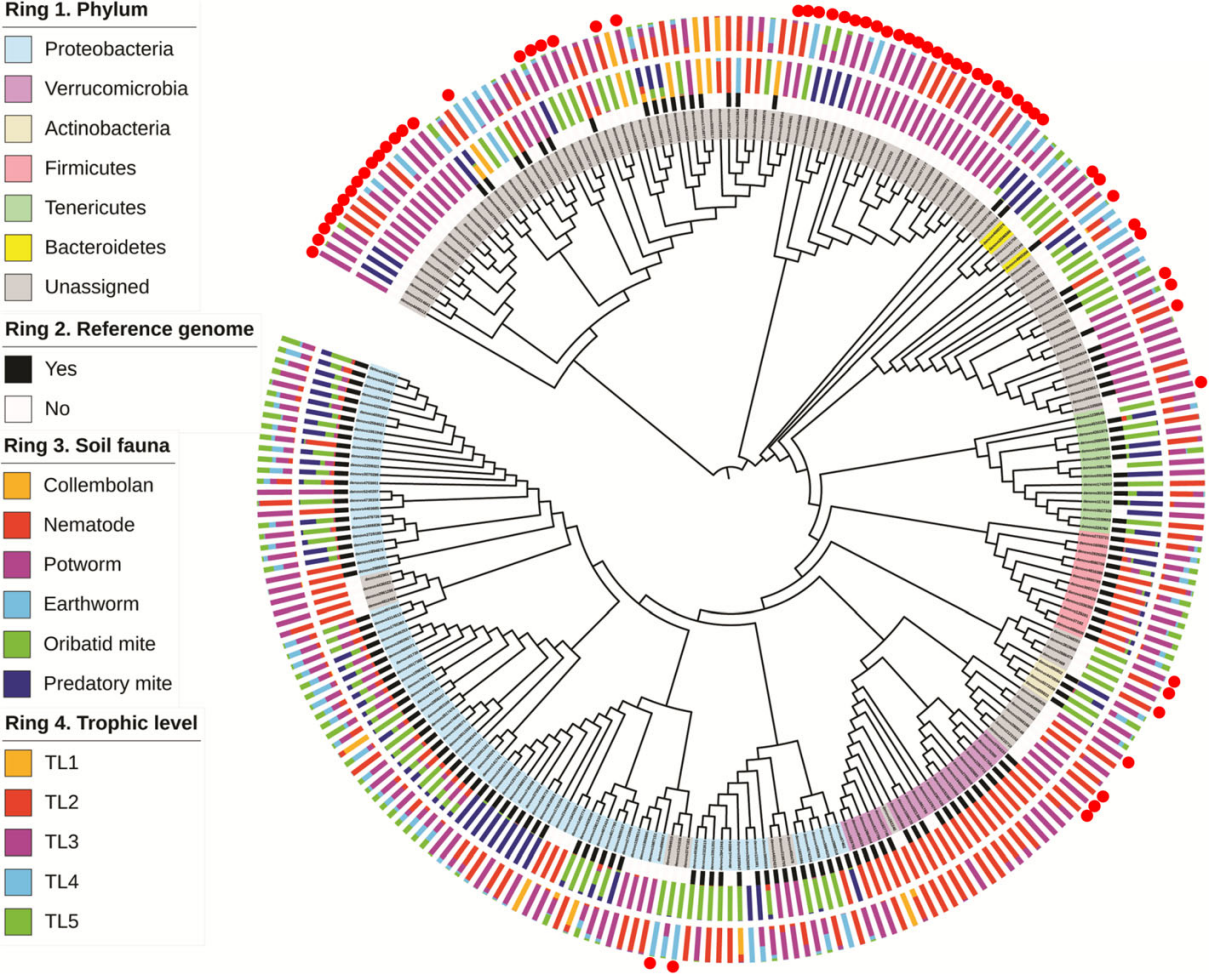
Phylogenetic distribution of the most abundant 258 unique bacterial taxa in the soil fauna microbiome. The innermost ring 1 indicates microbial classification at the phylum level. Black shading on ring 2 indicates for each bacterial taxa whether there is a representative isolate and a genome match at the ≥ 97% 16S rRNA gene sequence similarity level. Rings 3 and 4 indicate the relative proportion of each bacterial taxa in different soil fauna microbiomes and different trophic level of animals, respectively. We normalized the difference in relative abundance across all the OTUs based on the total relative abundance of each bacterial taxa across all the samples. The “TL” indicates the trophic level of soil fauna based on natural 15N fractionation of animal body tissue. The red dots indicate that unassigned taxa with no genome representatives were exclusively found in potworms, oribatid mites, and predatory mites

**Fig. 6:**
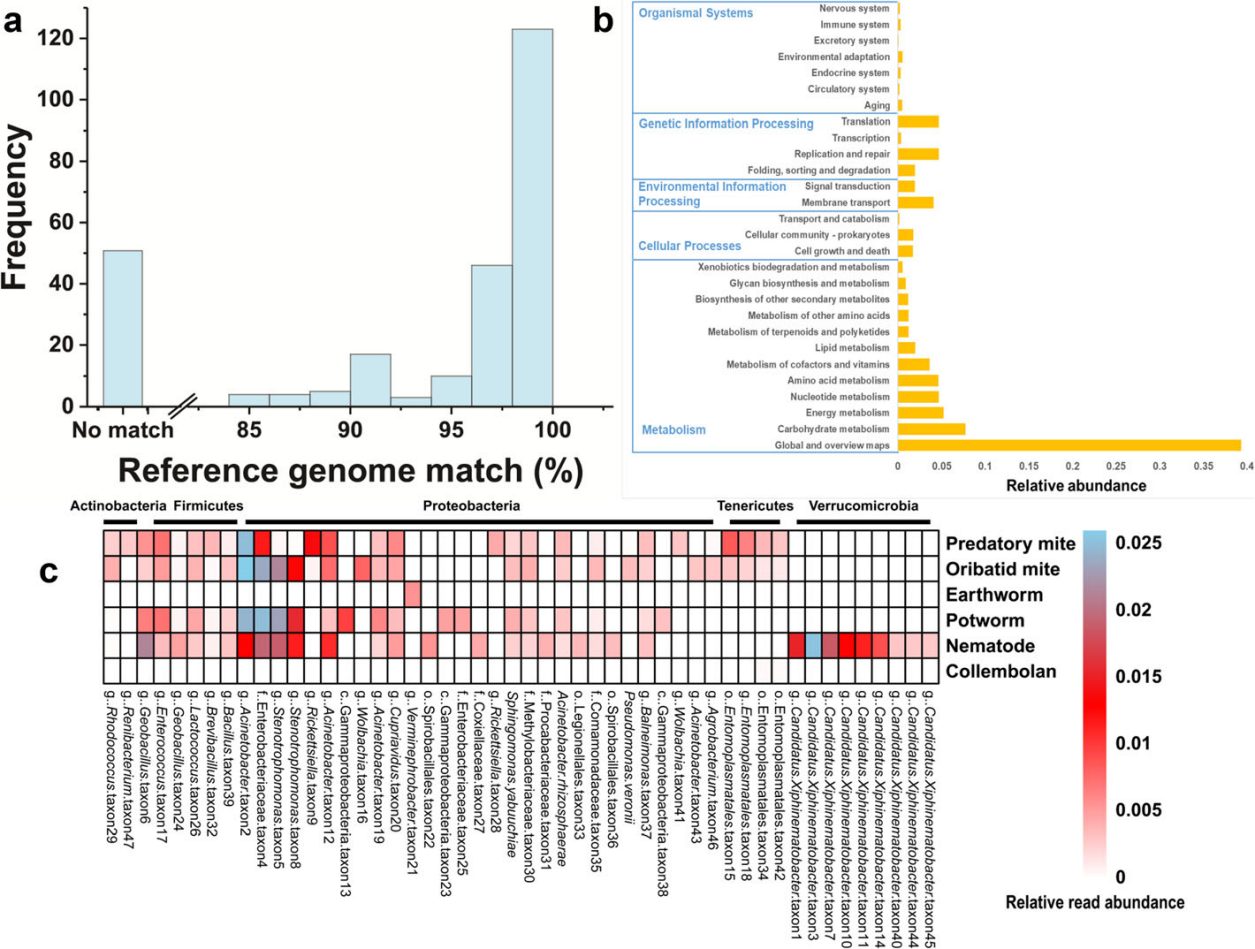
**a** Histogram revealing the percentage 16S rRNA gene sequence similarity between the most abundant 258 unique bacterial taxa and the most closely related available reference genome for each bacterial taxon. The “No match” means that the reference genome was not found in the NCBI database. **b** The functional profiles of the uncharacterized taxa (“no match”) was predicted by the Tax4Fun2. **c** Heatmap revealing the read abundance of the 50 most abundant unique bacterial taxa in different soil fauna microbiomes

Microbial diversity (*R*^2^ = 0.826, *P* < 0.001; Fig. 4a) and predicted microbial functional diversity (*R*^2^ = 0.256, *P* < 0.001; Fig. 4b) (Shannon index) had a positive significant correlation with the δ ^15^N value of soil fauna body tissues (trophic level), respectively. The number of unique microbial taxa was lowest in collembolans and earthworms, intermediate in nematodes and potworms, and highest in oribatid and predatory mites (Fig. 4c). Further, the number of the unique microbial taxa increased with the hierarchical position of the host within the food web (Fig. 4d). A phylogenetic tree of the most abundant 258 unique bacterial taxa (maximal read abundance > 5%) in the soil fauna microbiome was constructed (Fig. 5). Only 62% of these taxa had a genome match in the NCBI database (> 97% 16S rRNA identify). We observed that all unique microbial taxa belong to *Verrucomicrobia* were exclusively found in nematodes, all unique microbial taxa belong to *Bacteroidetes* were only determined in nematodes, oribatid mites, and predatory mites, most of unique microbial taxa belong to *Actinobacteria* and *Tenericutes* were exclusively observed in oribatid mites and predatory mites, and many unique microbial taxa belong to *Proteobacteria* were only detected in nematodes, potworms, oribatid mites, and predatory mites. Some of these abundant and unique bacterial taxa were also reported in previous studies [12–15, 19]. A collection of unassigned taxa with no genome representatives were exclusively found in potworms, oribatid mites, and predatory mites, which were highlighted by red dots in Fig. 5. The majority of unmatched taxa had no cultivated representatives (Fig. 6a). The Tax4Fun2 prediction showed a total of 28 KEGG systems in the functional profile of the uncharacterized taxa (“no match”), which were involved in metabolism, cellular processes, environmental information processes, genetic information processing, and organismal systems (Fig. 6b). The heatmap showed that the read abundance of most of the unique microbial taxa was lower in collembola and earthworms than in the other soil fauna (Fig. 6c). We also investigated changes in the relative abundance of the 23 dominant, prevalent, and unique bacterial taxa across trophic levels (maximal read abundance > 1% and those found in more than 50% of soil fauna samples) using a heatmap diagram (Fig. S21). The 65% of these unique bacteria showed significant enrichment in the soil faunal microbiome as trophic level increased (*P* < 0.001; Figs. S21-S22). The similar enrichment pattern was also observed by visualizing the dataset based on each soil faunal group (Fig. S23).

**Fig. 7:**
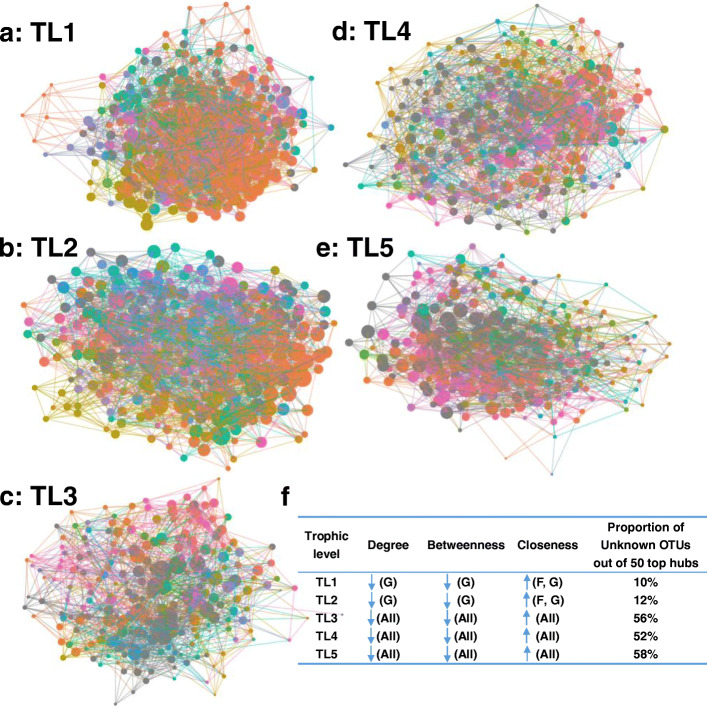
Hub analysis of microbial networks from different trophic level of soil fauna microbiome (**a**–**e**). Soil fauna microbial networks at the genus level with nodes sized as a function of hub score. Nodes are colored by genus classification with unknown taxa depicted in dark gray. **f** Effects of the removal of unknown OTUs from the network on network metrics. Downward pointing arrow indicated significant decrease and upward pointing arrow indicated significant increase. F family, G genus, and all all taxonomic levels

Network analysis showed that more unknown OTUs occurred as top hubs with an increase in the trophic level (Fig. 7), suggesting that microbial dark matter had a more important ecological role in the microbiome of the higher trophic levels of soil fauna. The removal of unknown OTUs significantly reduced values of degree and betweenness and increased closeness in TL3, TL4, and TL5 network metrices across all taxonomic levels but TL1 and TL2 only at the genus level (*P* < 0.05; Figs. 7f and S24). Only in the TL5 network metric, the degree centrality score of without unknown taxa network was distinctly lower than that in the bootstrap network at the genus level (*P* < 0.001; Fig. S24).

## Discussion

### Hidden microbial diversity in the soil food web

Here, we present a systematic characterization of the microbiome of six functionally important soil faunal groups from diverse ecosystems across China. Significant differences between the microorganisms in the soil fauna and the surrounding soil were observed in all samples and at each sampling site, respectively. This suggests that there is selective acquisition and colonization of soil faunal microbiomes and that they represent a unique and diverse set of microbial niches. This could be because the microbial habitat available inside soil fauna (e.g., high nutrient content and relatively anaerobic) is different from the surrounding environment [12, 56], as confirmed in previous studies involving fruit flies [39, 50], nematodes [12], collembolans [13], and beetles [57]. Different types of soil fauna commonly have different diets [29, 30], and this might also contribute to differences in their microbial communities. Interestingly, we found the collembolans and earthworms sharing more similar microbial communities to soils compared to the other soil fauna, which may be related to their habit. Earthworms tend to feed on soil, which may make their microbes similar to soil. The ecdysis of collembolan along with its intestines decreases host selection for microbiota and makes the collembolan microbiomes more susceptible to the soil environment.

In the present study, the analysis of additive diversity partitioning showed that soil faunal individual and group contributed most to microbial diversity. The microbiome of each soil faunal individual contained more diverse and abundant microbiota than that reported from other invertebrates, such as honey bees [58], fruit flies [39], or caterpillars [59]. Considering that soil fauna themselves are highly diverse [1, 3, 4], and their microbiome is similarly diverse, soil fauna are likely to be a hidden pool of microbiota. Our findings strongly suggest that soil fauna contain multiple, unique microbial species in their microbiomes which are rare, or not detected, in the more general soil environment. Consequently, they could represent a valuable resource for bioprospecting because interesting microbial compounds such as antibacterial agents can be recovered from soil faunal microbiomes [60, 61].

These unique microbial taxa may arise from unidentified environmental sources or inhabit a micro-environment [50] such as water films around soil particles that soil animals disproportionately consume. Alternatively, these bacteria may persist and thrive solely within soil fauna. This latter explanation is circumstantially supported by our findings that host species had a strong selective influence on the microbiome of each faunal group. This further indicates that soil animals are important as repositories of microbial biodiversity. The existence of this microbial niche was largely unknown prior to the present study and raises the issue that the holobiont of soil fauna should be considered as a target of conservation [62, 63].

Our results showed that sampling sites also had an important contribution to the composition and diversity of soil faunal microbiome, suggesting that environmental change might affect the diversity of soil faunal microbiome. In the present study, landuse significantly altered the diversity of soil faunal microbiome, which also confirmed this viewpoint. A recent study showed that anthropogenic climate warming altered animal microbiomes [64]. Thus, more attention should be focused on potential changes to soil faunal microbiomes under global change. The other thing we have to admit is that this study represents a single snapshot in time. As suggested, if generalist feeding results in high microbiome diversity, then other dimensions of the generalist diet need to be studied in the future. For instance, how much would our results change if we have collected samples over multiple time points to capture seasonality in diet? In addition, the analysis of enterotype (not directly related with host or environmental factors) revealed that each soil faunal group all had enterotypes dominated by the genus *Acinetobacter*, a common opportunistic pathogen [65]. Compared with a previous study [12], we found that dominant bacteria in the enterotype of wild nematodes are different from laboratory-reared nematodes. These suggest that the dominant bacteria in the soil faunal microbiome may be controlled by a host-specific enrichment process and environmental selection.

### Ecological role of microbial dark matter in the soil food web

The co-occurrence of diverse unknown taxa in the soil food web microbial network was observed in the present study. This suggests that these microbial taxa may be phylogenetically related, similar to classified OTUs [66, 67]. We found that unknown taxa significantly affected values of betweenness and degree in soil faunal microbial networks, indicating that unknown taxa could influence microbial interactions. Moreover, many unknown OTUs occurred as top hubs in microbial networks in the soil food web and in each soil faunal group. Because hubs significantly contribute to network structure and cohesiveness, they are important parts of networks [20]. The potential functions of these taxa are also unknown, and consequently microbial dark matter plays an unknown but potentially important ecological role in the soil faunal microbial network.

### Contribution of neutral processes to the assembly soil faunal microbiome

In the present study, we found that the predictive ability of a neutral model was better than the binomial distribution, which suggested that the processes of passive dispersal and ecological drift might contribute to soil faunal microbial community assembly. This point was quantitatively confirmed in the results of phylogenetic bin-based null model and consistent with other invertebrates [39, 50]. The present findings support our hypothesis that more OTUs could be explained by the neutral processes in microbiomes of nematode, potworm, and earthworm compared to collembola, oribatid mites, and predatory mites, suggesting that the microbiomes of nematode, potworm, and earthworm were more difficult to predict using environmental data. This may be related to the feeding behavior of soil fauna [29]. Collembola, oribatid mite, and predatory mite have a wider selection of food than the nematode, potworm, and earthworm [26, 27, 68]. Thus, the selected feeding behavior may restrict random sampling and immigration of OTUs within the collembolan, oribatid mite, and predatory mite. Our results indicated that landuse could affect the relative importance of stochastic processes to the assembly of soil faunal microbiome, and more stochastic processes were found in the farmland samples. Because resources are more homogenized in the farmland due to the reduction of plant diversity compared to the forest [69], this may lead to an increase of randomness in the soil animal feeding. This suggests that soil animal microbiome is more easily changed in the farmland ecosystem.

### Trophic dynamics of host microbiome in the soil food web

The positive relationship between trophic level and microbial biodiversity (Shannon index), unique microbial taxa, and ecological role of microbial dark matter was observed in the present study, which has not been described before. This result suggests that soil fauna at the top of the food web harbor more diverse and unique microbiomes. The reason for this may be because predators in soil food webs might be generalists [29, 30], which could ingest multiple low trophic organisms. This means that, apart from the environment, they could acquire microbiota from multiple low trophic organisms, and some microbes unique to different low trophic organisms might coexist within predators. Therefore, the predator might have a more diverse and unique microbiome compared to a lower trophic organism. The result of FEAST analysis confirmed the point that the predatory mite (top predator) has diverse microbial sources. Alternatively, our result showed that different trophic level of soil fauna shared most OTUs, suggesting that microbes can move between trophic levels (prey-predator) [70] and become enriched at higher trophic levels. Finally, we found that deterministic processes played an important role in the microbial assembly of soil food web, suggesting that deterministic processes might contribute to the assembly of unique microbiota in soil fauna due to a host-specific enrichment process [50].

It has been recognized that host microbiome can be used to predict disease and age of humans [71–73]. Because of the complexity and invisibility of the soil food web [9, 30], the determination of trophic level of soil fauna is always the frontier and at the highest level of difficulty in soil ecology [29]. In the present study, we identified that the trophic level of animals in the soil food web could be predicted by analyzing microbiomes, which will be useful for soil ecology studies. This could be because the animal microbiota might be a reflection of diet. Since diet determines trophic level in the food web [29, 30], microbiota might be used as a predictive tool.

### Global implications of soil faunal microbiome

The soil faunal microbiome is an important component of the global microbiome and contributes to global biodiversity [12, 13, 56, 70]. Recent studies have showed that top predators are more vulnerable to global change and that their influence on food web interactions is more important than their biomass suggests [8, 10, 11]. Consequently, the loss or extinction of these soil fauna will have multiple effects. Some of these effects are predictable, such as significant changes to nutrient and energy flows [74, 75], but our results suggest a hidden and unsuspected consequence (Scenarios 1 and 2; Fig. S1): the extinction of large numbers of unique microbial species and microbial dark matter. Higher trophic levels are a repository of a greater proportion of unique bacterial diversity, even for the most dominant taxa. At the same time, we also found that microbial dark matter in the higher trophic level of the soil faunal microbiome had a more important ecological role in the soil ecosystem. These hidden microbiotas are likely to rely on their multicellular hosts for survival and potential functions [76, 77], and preservation of key soil invertebrates, particularly those at the top of the food web, will also preserve the hidden and unique microbial biodiversity within their microbiomes.

Our results further provide evidence that functional and taxonomic information is largely lacking for the unique bacterial taxa residing in soil fauna, especially for fauna at the top of the food web. Since these unique bacterial taxa are also different between different types of soil invertebrates, this further indicates that soil fauna harbor diverse and unique assemblages of microorganisms. Recent studies have documented the silent extinctions of soil invertebrates on a global scale [8, 11]. Our study suggests that losses of even a single soil invertebrate species could result in the loss of numerous unique microbial taxa about which we know very little. Such losses could also cascade into permanent changes in ecosystem function and resilience, because in the present study, we found that these unique bacterial taxa play an important role in microbial networks. Protecting soil invertebrates would simultaneously protect the multiple species living in their microbiomes [17]. By protecting these unique microbial taxa we also protect their genetic resources and potential ecological functions, both for ecosystem health and for potential discovery of biological tools in the future [60, 61, 78].

## Conclusions

Soil faunal microbiomes contain highly diverse and unique microbial taxa, with microbial dark matter playing an important ecological role in the soil faunal microbial network. Stochastic and deterministic processes all play important roles in the microbial assembly in soil food webs. This microbial diversity, the unique microbial taxa, and the ecological roles of microbial dark matter all increase with hierarchical position of the host within the food web. This suggests that the extinction of single-soil invertebrate species, especially those at the top of the food web, will result in extinctions of the microbial communities living in their microbiomes. This ecological perspective and the hidden biodiversity have not been fully considered in biodiversity and conservation debates. Mass extinctions of this hidden microbial diversity could have serious and unpredictable consequences for the functioning of Earth systems.

## Supplementary Information

**Additional file 1: Supplementary Text.** Characterization of microbial community in the soil food web; **Table S1.** The information of used primers of the DNA barcoding; **Table S2.** Comparison of soil fauna microbiome composition using PERMANOVA (Adonis test); **Table S3.** Effects of the removal of Unknown OTUs from the network on network metrics; **Figure S1.** The potential relationship between the loss of microbial species living within the microbiome of soil fauna and soil faunal extinction; **Figure S2.** The distribution of sample sites across China based on different climatic zones (A suffix of 1 in the location indicates farmland, while 2 indicates forested land); **Figure S3.** The relative abundance of 18 most abundant bacterial families (a) and 16 most abundant bacterial species (b) in all samples, classified by sample types; **Figure S4.** The alpha diversity of soil and fauna microbial communities at a sequencing depth of 13551; **Figure S5.** Principal coordinates analysis (PCoA) revealing the distribution of soil faunal bacterial communities using the weighted unifrac distance in each sampling site; **Figure S6.** Principal coordinates analysis (PCoA) revealing the distribution of soil faunal bacterial communities using the unweighted unifrac distance in each sampling site; **Figure S7.** Shared OTUs between soil and soil fauna; **Figure S8.** Principal coordinates analysis (PCoA) revealing the distribution of soil faunal bacterial communities using the weighted unifrac distance in each soil faunal group; **Figure S9.** Principal coordinates analysis (PCoA) revealing the distribution of soil faunal bacterial communities using the unweighted unifrac distance in each soil faunal group; **Figure S10.** The PERMANOVA analysis revealing the relative contribution of landuse, sampling site and soil faunal species to the variation of each soil faunal group microbiome; **Figure S11.** Enterotyping (clustering) of each soil faunal group, which was clustered using the Jensen–Shannon distance and partitioning around medoids method based on the relative abundance of bacterial genera; **Figure S12.** Analysis of microbial network taxa interconnectedness from the soil food web microbiome; **Figure S13.** Network analysis revealing microbial taxa interconnectedness at the Genus level; **Figure S14.** Boxplots presenting the difference of betweenness, closeness, and degree centrality values of nodes between the three network types (Original-Without Unknown, Original-Bootstrap and Without Unknown-Bootstrap) at different taxonomic levels; **Figure S15.** Fit of neutral model for collembola (a), nematodes (b), potworms (c), earthworms (d), oribatid mites (e) and predatory mites (g); **Figure S16.** Boxplot revealing natural ^15^N fractionation (δ ^15^N value) of different soil fauna (centre line, median; box limits, first and third quartiles; whiskers, 1.5 × interquartile range); **Figure S17.** FEAST estimations of source contribution to the sink (predatory mite) in each sampling site; **Figure S18.** Venn diagram revealing the shared OTUs number between different trophic level of soil fauna; **Figure S19.** Change in relative read abundance of 26 dominant bacterial taxa along an increase in trophic level (δ ^15^N value) of the soil fauna; **Figure S20.** Random forest classification of soil fauna microbial communities (including 58 bacterial taxa which were found in more than 70% of all soil fauna samples) based on different animal types (a and b) and trophic levels (c and d); **Figure S21.** Changes in relative read abundance of 23 unique bacterial taxa across increased trophic level as measured by δ ^15^N value (-0.25-16.79) in the soil fauna; **Figure S22.** Linear fitting diagrams revealing unique bacterial taxa that enriched with increasing δ ^15^N value (-0.25-16.79) in the soil fauna (P < 0.001); **Figure S23.** Scatter diagrams revealing changes in relative read abundance of 23 unique bacterial taxa across increased trophic level as measured by δ ^15^N value (-0.25-16.79) in the soil fauna; **Figure S24.** Boxplots presenting the difference of betweenness, closeness, and degree centrality values of nodes between the three network types (Original-Without Unknown, Original-Bootstrap and Without Unknown-Bootstrap) at the Genus level, which reflected effects of Unknown taxa on different trophic level soil faunal network metrics.

**Additional file 2.** The detailed information for each sample (e.g. geographic location, sampling date, host classification), and the description of how our soil fauna samples correspond to each of the five fauna samples collected per site.

**Additional file 3.** A diagram of the hierarchy based on the current knowledge of soil invertebrates revealing the relationship among collembola, nematode, potworm, earthworm, oribatid mite and predatory mite in the food web.

**Additional file 4.** The R scripts.

## Data Availability

We have deposited all sequencing data into the NCBI Sequence Read Archive under BioProject accession number PRJNA511675 and supplied the detailed information for each sample (e.g., geographic location, sampling date, host classification) in the Additional file 2. The authors declare that the other main data supporting the findings of this study are available within this article and in the Additional files. Extra data supporting the findings of this study are available from the corresponding author upon reasonable request. Custom codes for all analyses are available from the Additional file 4.
