# Supplementary material for: Trophic level drives the host microbiome of soil invertebrates at a continental scale
Source: Microbiome. 2021 Sep 20;9:189. doi: 10.1186/s40168-021-01144-4 (PMC8454154; doi:10.1186/s40168-021-01144-4)
Supplement: Supplementary file 2 — Additional file 1: Supplementary Text. Characterization of microbial community in the soil food web; Table S1. The information of used primers of the DNA barcoding; Table S2. Comparison of soil fauna microbiome composition using PERMANOVA (Adonis test); Table S3. Effects of the removal of Unknown OTUs from the network on network metrics; Figure S1. The potential relationship between the loss of microbial species living within the microbiome of soil fauna and soil faunal extinction; Figure S2. The distribution of sample sites across China based on different climatic zones (A suffix of 1 in the location indicates farmland, while 2 indicates forested land); Figure S3. The relative abundance of 18 most abundant bacterial families (a) and 16 most abundant bacterial species (b) in all samples, classified by sample types; Figure S4. The alpha diversity of soil and fauna microbial communities at a sequencing depth of 13551; Figure S5. Principal coordinates analysis (PCoA) revealing the distribution of soil faunal bacterial communities using the weighted unifrac distance in each sampling site; Figure S6. Principal coordinates analysis (PCoA) revealing the distribution of soil faunal bacterial communities using the unweighted unifrac distance in each sampling site; Figure S7. Shared OTUs between soil and soil fauna; Figure S8. Principal coordinates analysis (PCoA) revealing the distribution of soil faunal bacterial communities using the weighted unifrac distance in each soil faunal group; Figure S9. Principal coordinates analysis (PCoA) revealing the distribution of soil faunal bacterial communities using the unweighted unifrac distance in each soil faunal group; Figure S10. The PERMANOVA analysis revealing the relative contribution of landuse, sampling site and soil faunal species to the variation of each soil faunal group microbiome; Figure S11. Enterotyping (clustering) of each soil faunal group, which was clustered using the Jensen–Shannon distance and partitioning around [file 40168_2021_1144_MOESM2_ESM.docx]

Supplementary Text

**Characterization of microbial community in the soil food web**

Low abundance OTUs were removed, and individual samples rarefied to a depth of 13, 551 reads. In total, 33,248 OTUs from 46 bacterial phyla were analyzed in this study, generated from 9,892,230 high quality sequences. The 991 bacterial genera and 320 bacterial orders were represented by these OTUs, which mainly belonged to the phylum Proteobacteria (mean relative read abundance: 51.8%). Five dominant Bacterial families (Moraxellaceae: 17.5%, Enterobacteriaceae: 4.7% Xanthomonadaceae: 4.3%, Bacillaceae: 3.3% and Comamonadaceae: 3.2%) were identified in all samples, including soil and faunal samples (Fig. S3a). The *Acinetobacter* was the most common genus, being found in more than 97% of samples (Fig. S3b). Different soil fauna had different microbial species compositions, and these differed from the surrounding soil (Fig. S3). For example, enteric bacteria (Enterobacteriaceae) were commonly observed in nematode (5.7%), potworm (8.0%), earthworm (10.7%), orbatid mite (7.4%), predatory mite (4.5%) and collembolan samples (1.7%), but had much lower reads in soil samples (0.4%) (Fig. S3a). The dominant family Moraxellaceae (18.9%) was found in all soil faunal microbiomes, being highest in collembolan (35.4%), yet its relative read abundance was only 0.3% in soil samples (*P* < 0.05; Fig. S3a). Reads to the Hyphomicrobiaceae were common in soil (5.2%) and earthworm samples (4.9%) compared to all other samples (< 3%; *P* < 0.05). Of the most abundant taxa in soil faunal microbiomes, 16 of these taxa (22.6%) had their lowest observed abundance in soil (Fig. S3b). The alpha diversity of microbial communities (observed species and Shannon index) was significantly different between different types of fauna (*P* < 0.001; Fig. S4). The microbial community in soil had a higher Shannon index (9.41 ± 0.07) compared to the microbiota from soil faunal samples (*P* < 0.001; Fig. S4b). Overall, microbial diversity increased in the order of collembolan, nematode, potworm, earthworm, oribatid mite and predatory mite, with the greatest number of bacterial species (2868 ± 72) and the highest Shannon index (8.88 ± 0.13) being identified in predatory mite samples (Fig. S4; *P* < 0.001).

**Table S1** The information of used primers of the DNA barcoding

| **Species** | **Primer** | **Reference** |
| --- | --- | --- |
| Nematode | 5’segment of SSU | Floyd *et al*., 2002 |
| Potworm | H3 | Colgan *et al*., 1998 |
| Collembolan/ earthworm | COI | Orgiazzi *et al*., 2015 |
| Mite | 28S rDNA D3-1 | Fu Rong-Shu *et al.,* 2006 |

**Table S2** Comparison of soil fauna microbiome composition using PERMANOVA (Adonis test)

| Category | Weighted-Unifrac | | Unweighted-Unifrac | |
| --- | --- | --- | --- | --- |
|  | *P* | R^2^ | *P* | R^2^ |
| Host group | <0.001 | 0.82 | <0.001 | 0.25 |
| Host species | <0.001 | 0.87 | <0.001 | 0.38 |
| Host trophic level | <0.001 | 0.16 | <0.001 | 0.09 |
| Site | 0.017 | 0.02 | <0.001 | 0.02 |
| Landuse | 0.353 | <0.01 | 0.170 | <0.01 |

**Table S3** Effects of the removal of Unknown OTUs from the network on network metrics. Downward pointing arrow indicated significant decrease, and upward pointing arrow indicated significant increase. P: Phylum, F: Family, G: Genus and all: all taxonomic levels.

| **Faunal group** | **Degree** | **Betweenness** | **Closeness** | **Proportion of Unknown OTUs out of 50 top hubs** |
| --- | --- | --- | --- | --- |
| **Collembolan** | **(G)** | **(G)** | **(F, G)** | **16%** |
| **Nematode** | **(All)** | **(All)** | **(All)** | **52%** |
| **Potworm** | **(All)** | **(All)** | **(All)** | **60%** |
| **Earthworm** | **(G)** | **(G)** | **(F, G)** | **20%** |
| **Oribatid mite** | **(All)** | **(All)** | **(All)** | **46%** |
| **Predatory mite** | **(All)** | **(All)** | **(P to F)** | **52%** |
| **Soil food web** | **(All)** | **(All)** | **(G) (P to F)** | **40%** |


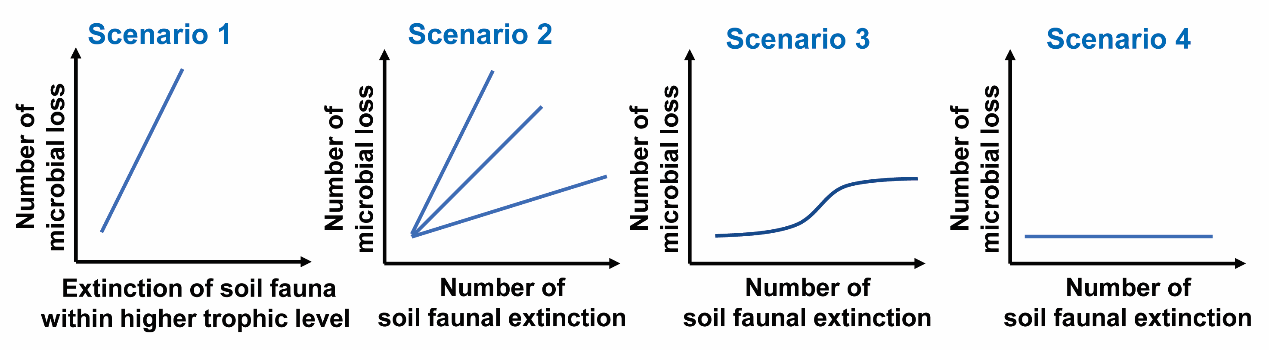


**Fig. S1. The potential relationship between the loss of microbial species living within the microbiome of soil fauna and soil faunal extinction.** Scenario 1: higher microbial diversity occurred in the higher trophic level of soil fauna with larger slope; Scenario 2: different soil invertebrates had different microbiomes and differed from environmental (e.g., soil) microbiomes, and different slopes indicated different trophic level; Scenario 3: different soil invertebrates shared similar microbiomes; Scenario 4: soil faunal microbiomes simply reflected the microbial communities in the environment (e.g., soil).


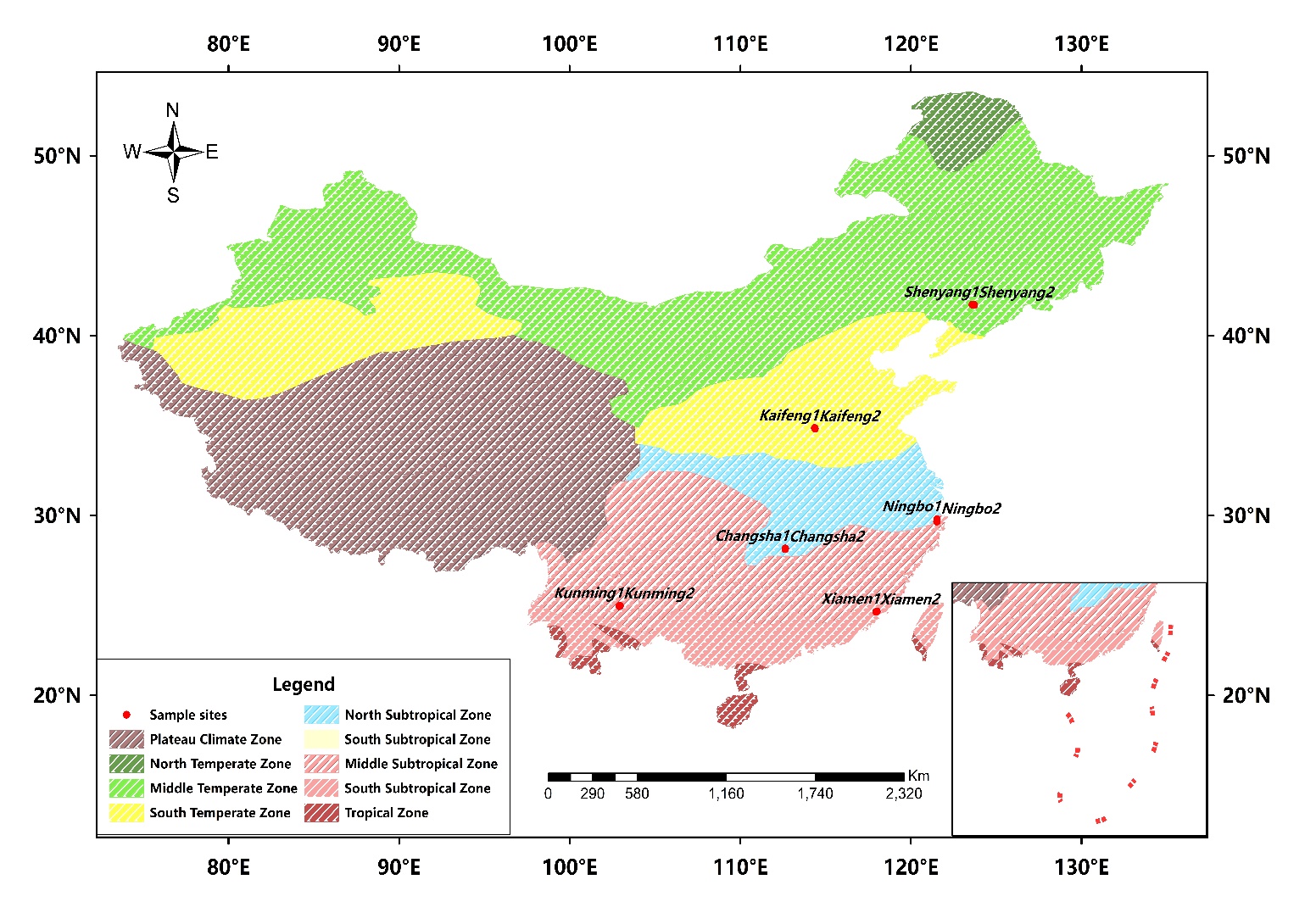


**Fig. S2.** The distribution of sample sites across China based on different climatic zones (A suffix of 1 in the location indicates farmland, while 2 indicates forested land).


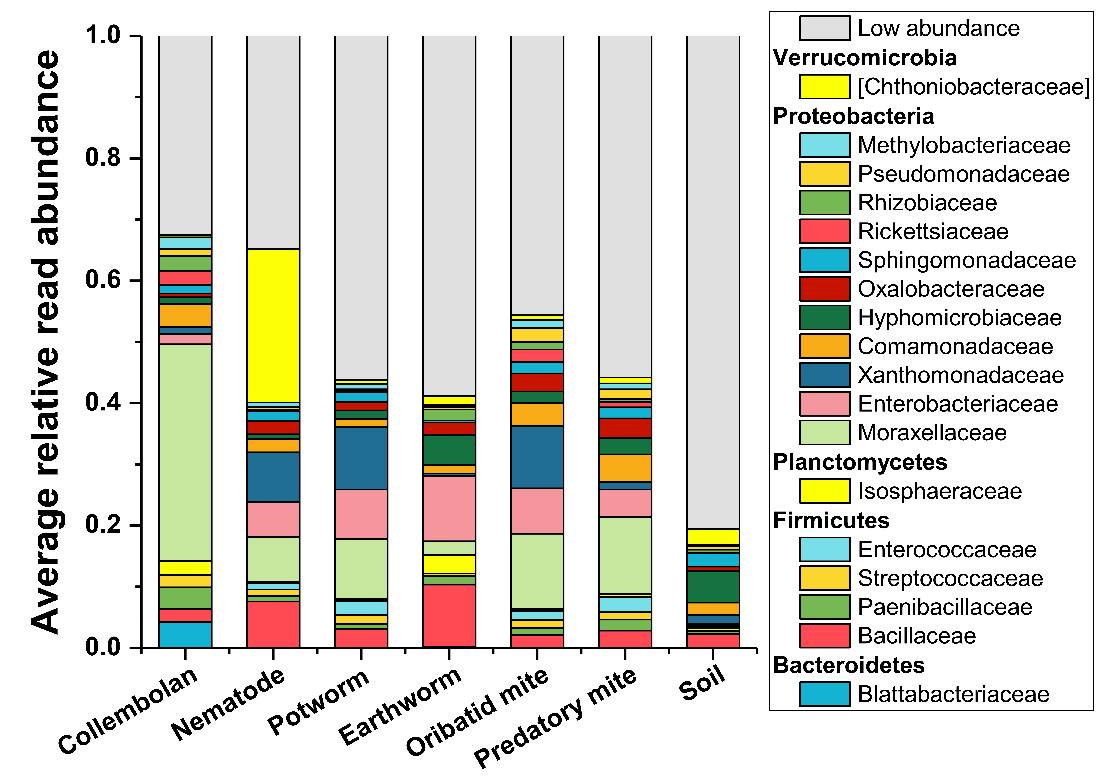


b

a


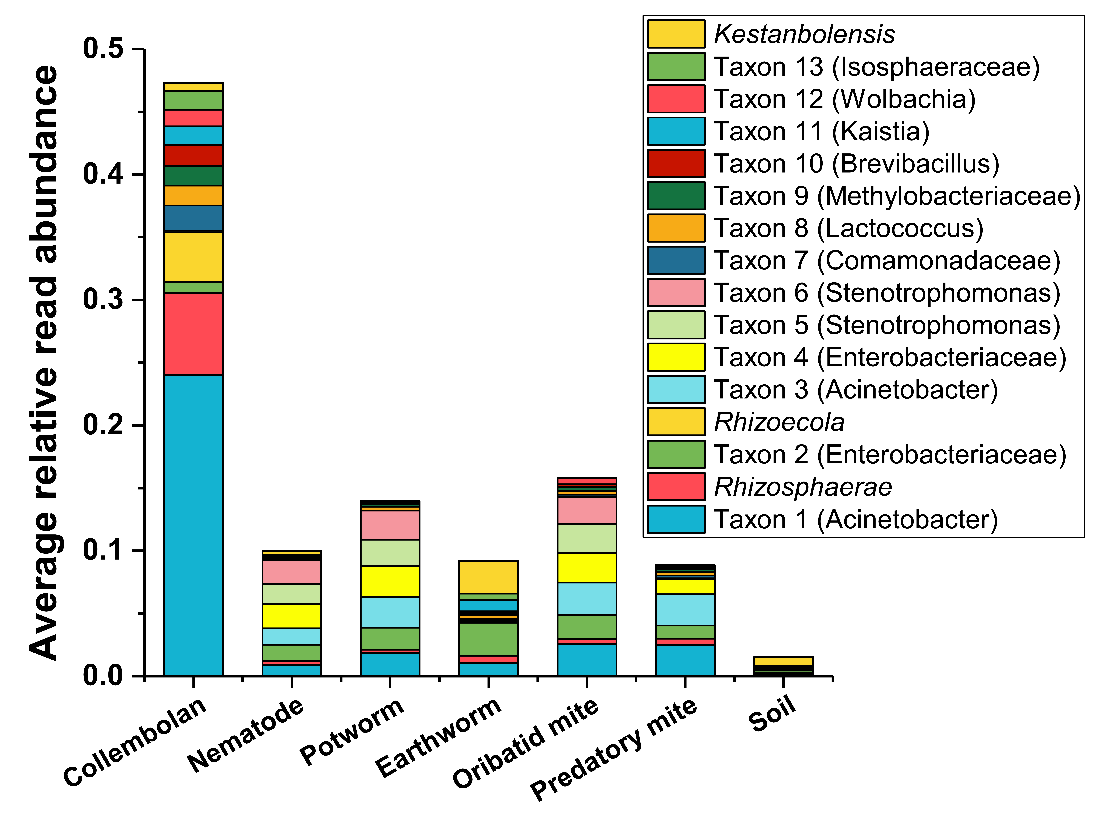


**Fig. S3.** The relative abundance of 18 most abundant bacterial families (a) and 16 most abundant bacterial species (b) in all samples, classified by sample types. The microbiota of low abundance were also characterized, but not shown in the figure.


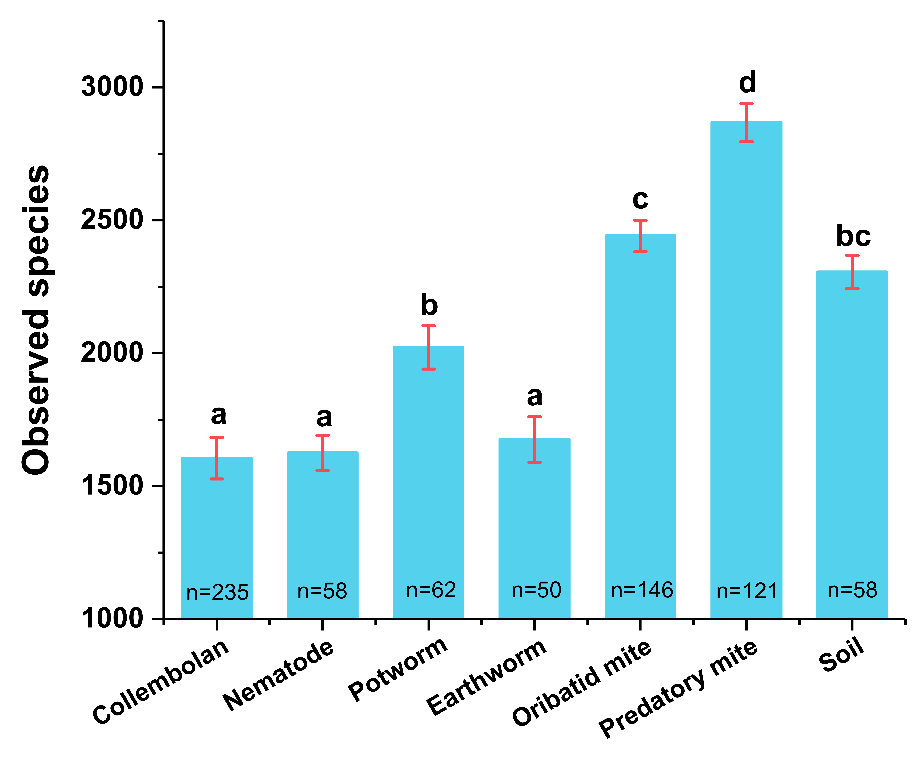


a


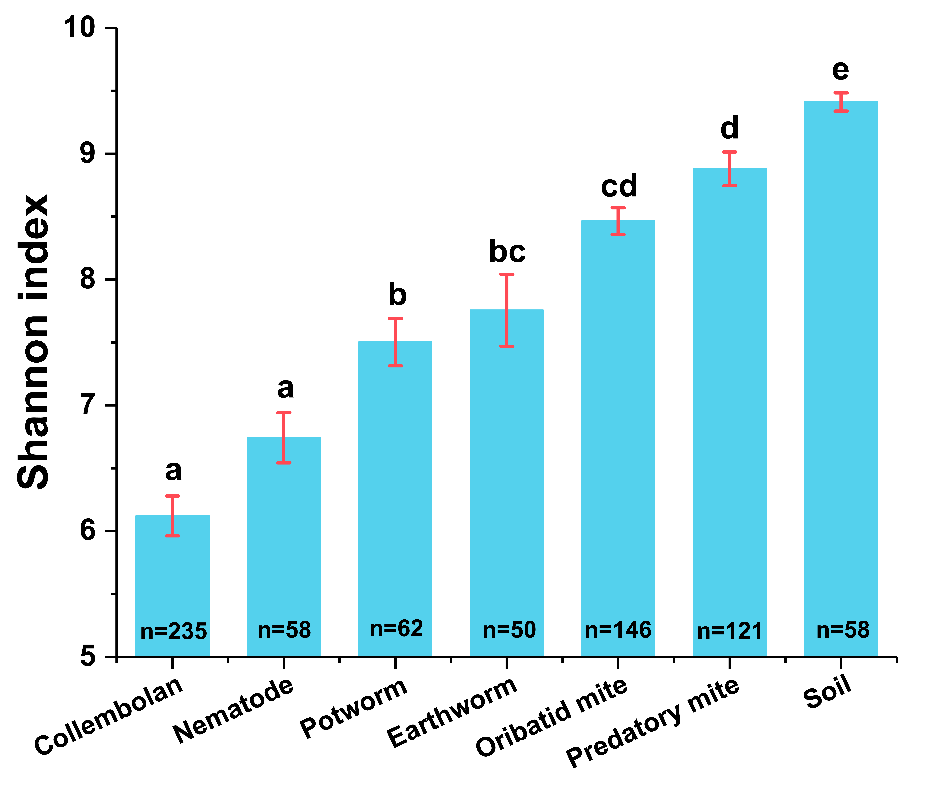


b

**Fig. S4.** The alpha diversity of soil and fauna microbial communities at a sequencing depth of 13551. The data are presented as mean ± standard error (SE). Since all data conformed to a normal distribution, ANOVA with the Duncan test was used to compare the difference in microbial diversity between different types of samples (Significance level: *P* = 0.05). Different letters indicate a significant difference between different types of samples.


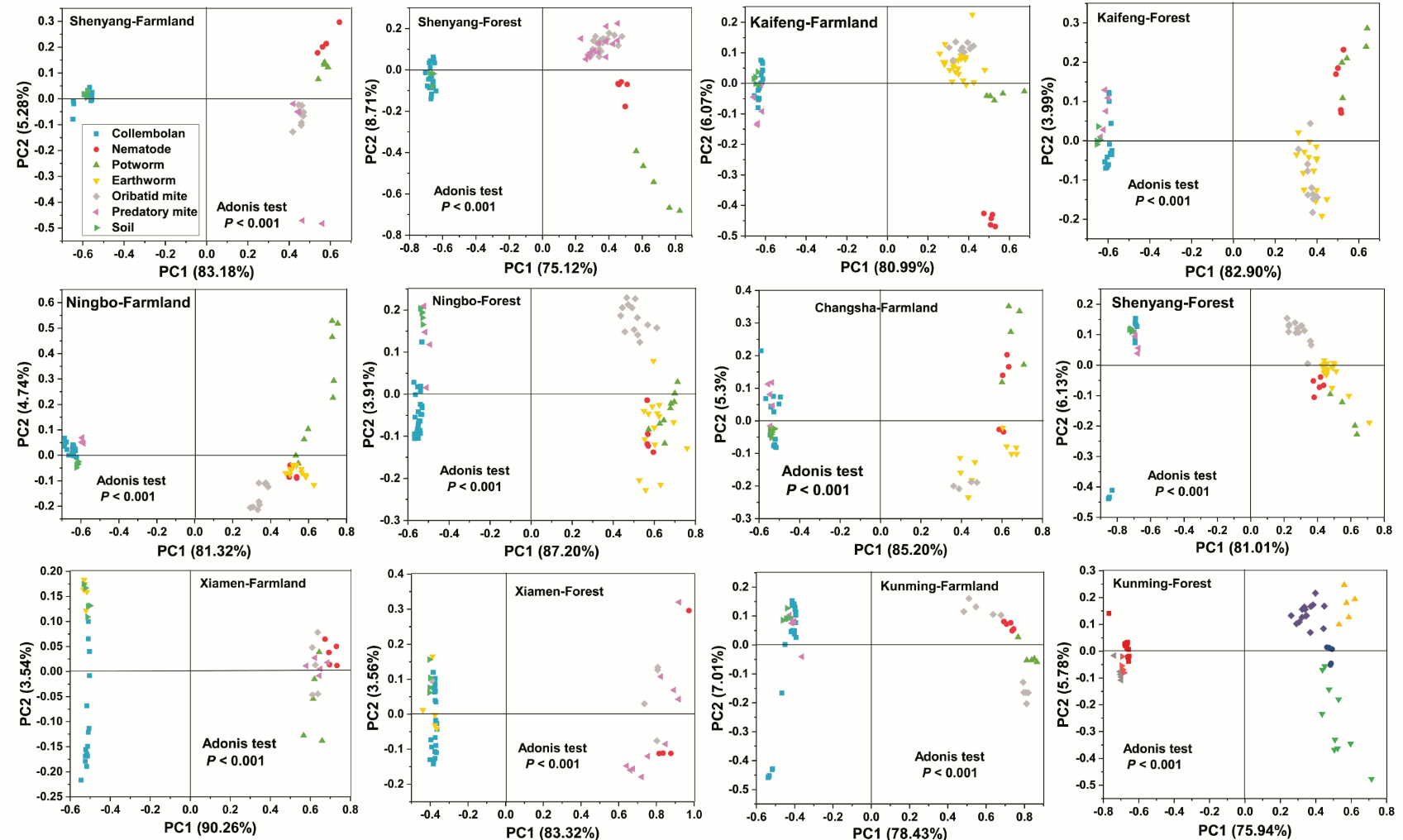


**Fig. S5.** Principal coordinates analysis (PCoA) revealing the distribution of soil faunal bacterial communities using the weighted unifrac distance in each sampling site. Different shapes and colors represented different soil faunal group.


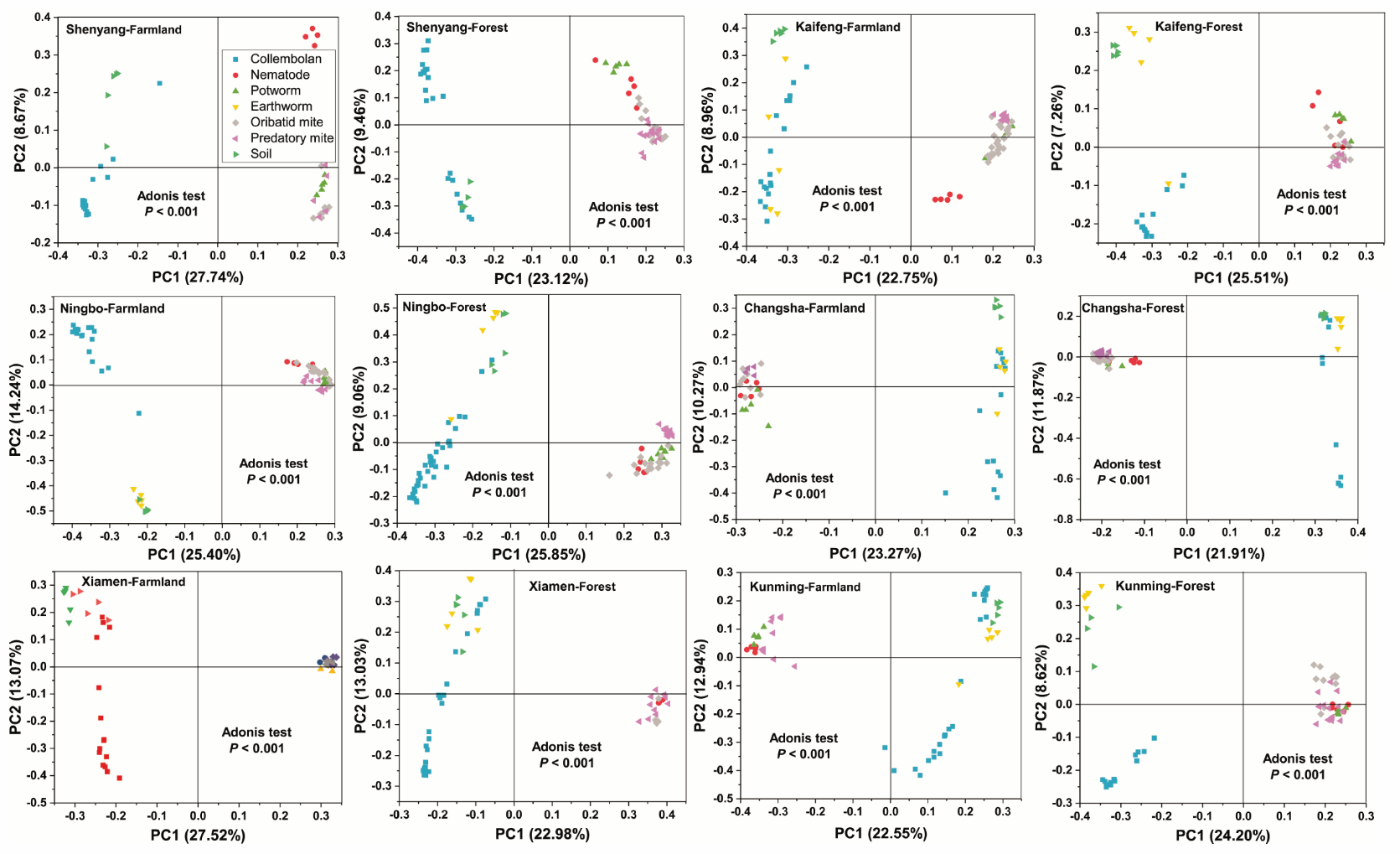


**Fig. S6.** Principal coordinates analysis (PCoA) revealing the distribution of soil faunal bacterial communities using the unweighted unifrac distance in each sampling site. Different shapes and colors represented different soil faunal group.


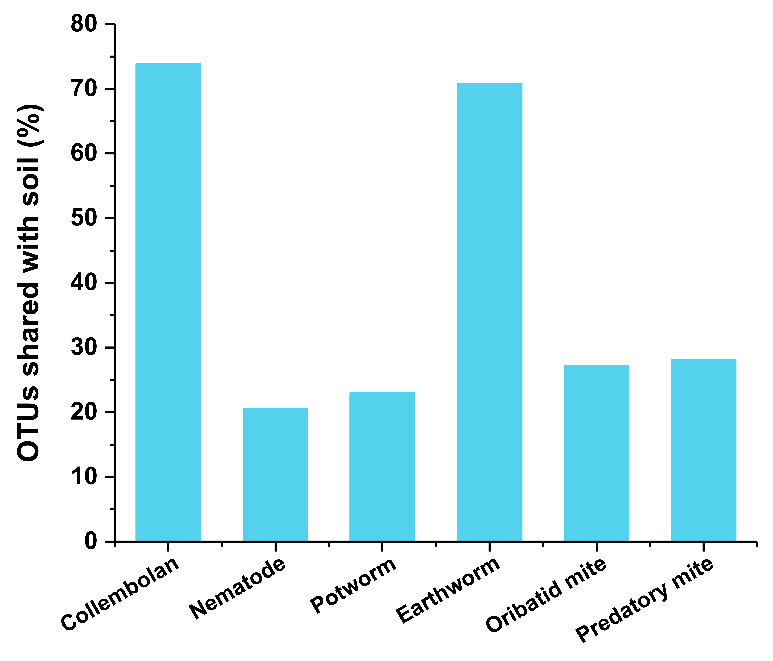


**Fig. S7.** Shared OTUs between soil and soil fauna.


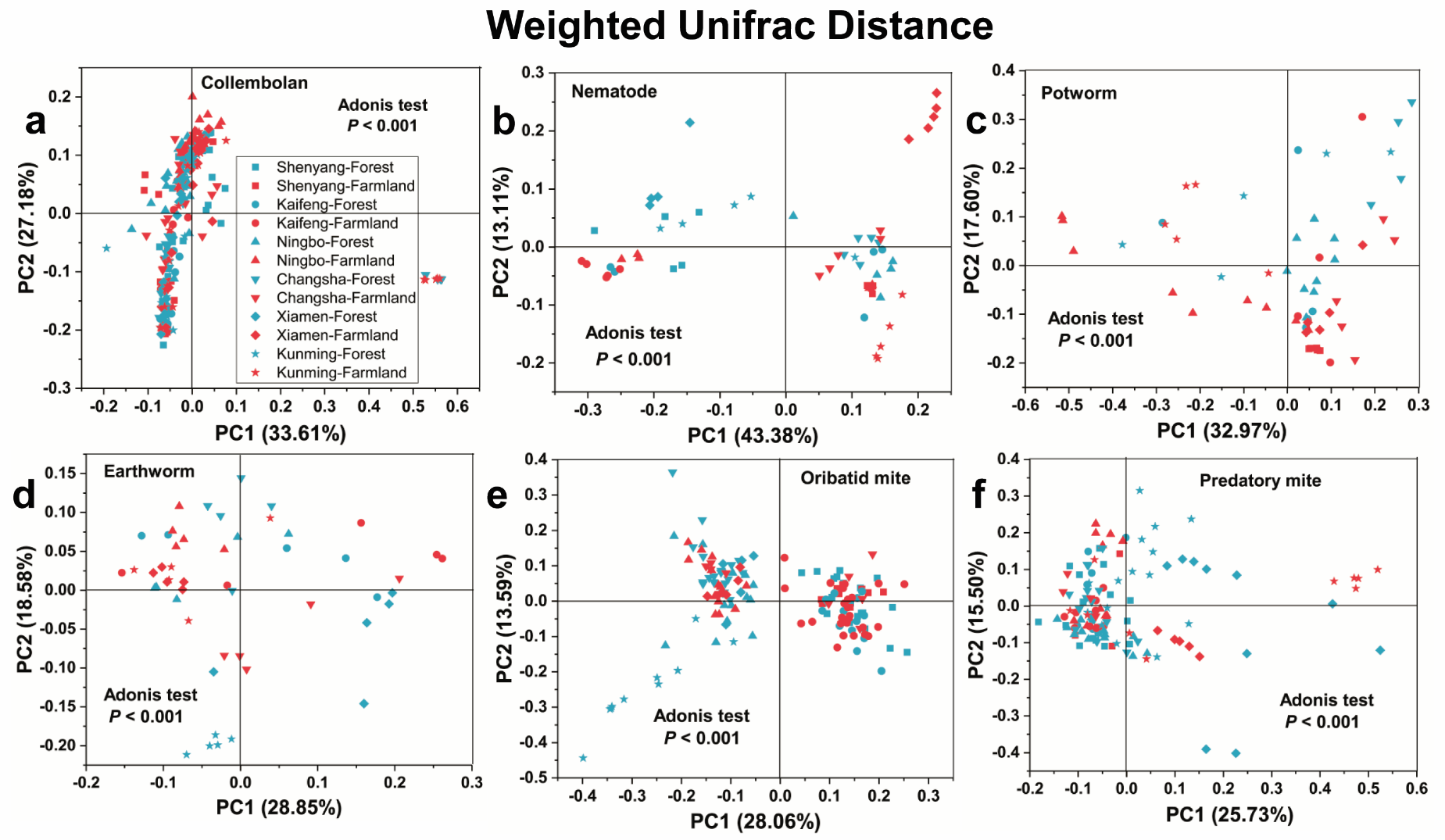


**Fig. S8.** Principal coordinates analysis (PCoA) revealing the distribution of soil faunal bacterial communities using the weighted unifrac distance in each soil faunal group. Different shapes represented different sampling sites, and different colors represented different landuses.


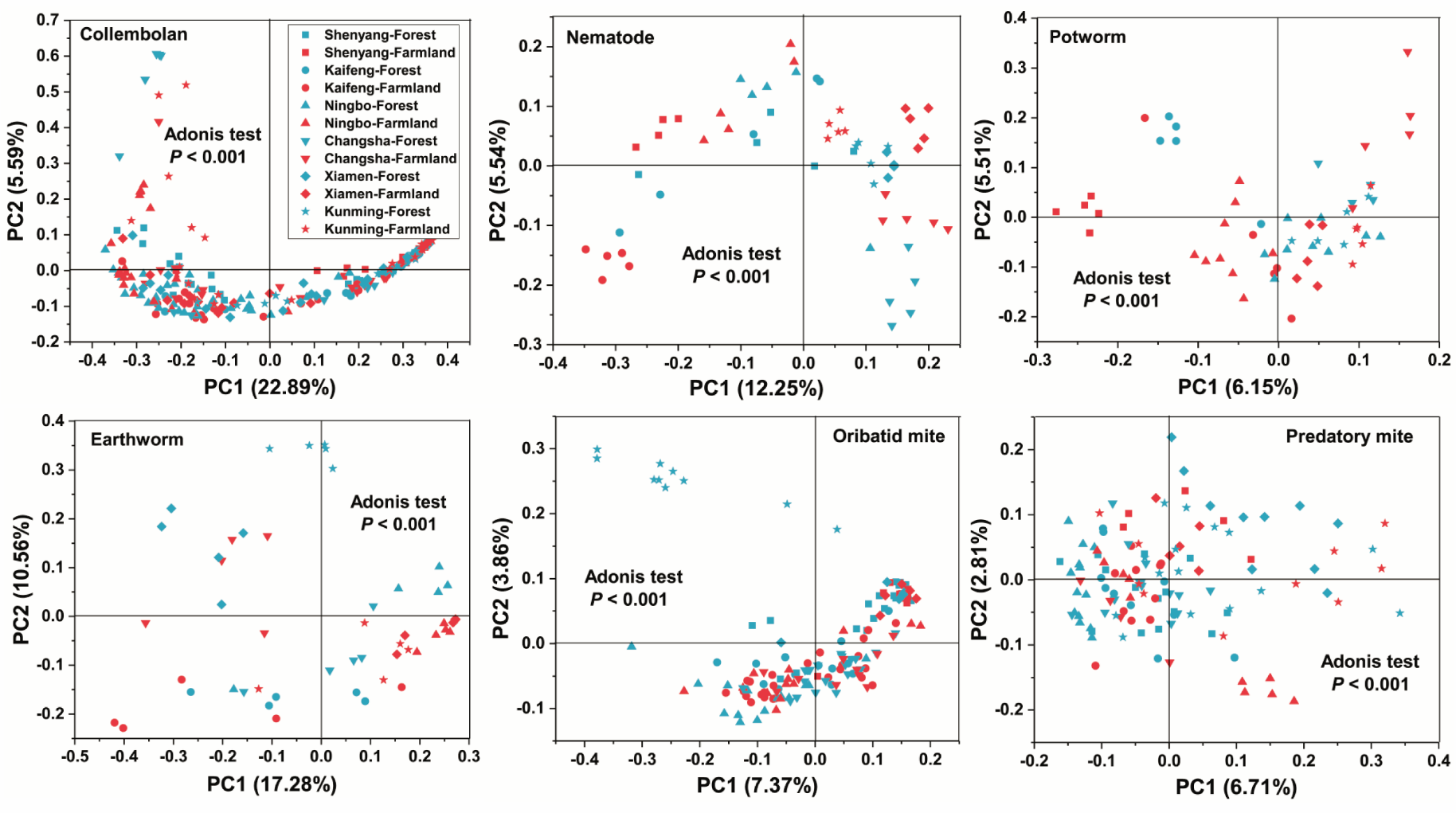


**Fig. S9.** Principal coordinates analysis (PCoA) revealing the distribution of soil faunal bacterial communities using the unweighted unifrac distance in each soil faunal group. Different shapes represented different sampling sites, and different colors represented different landuses.


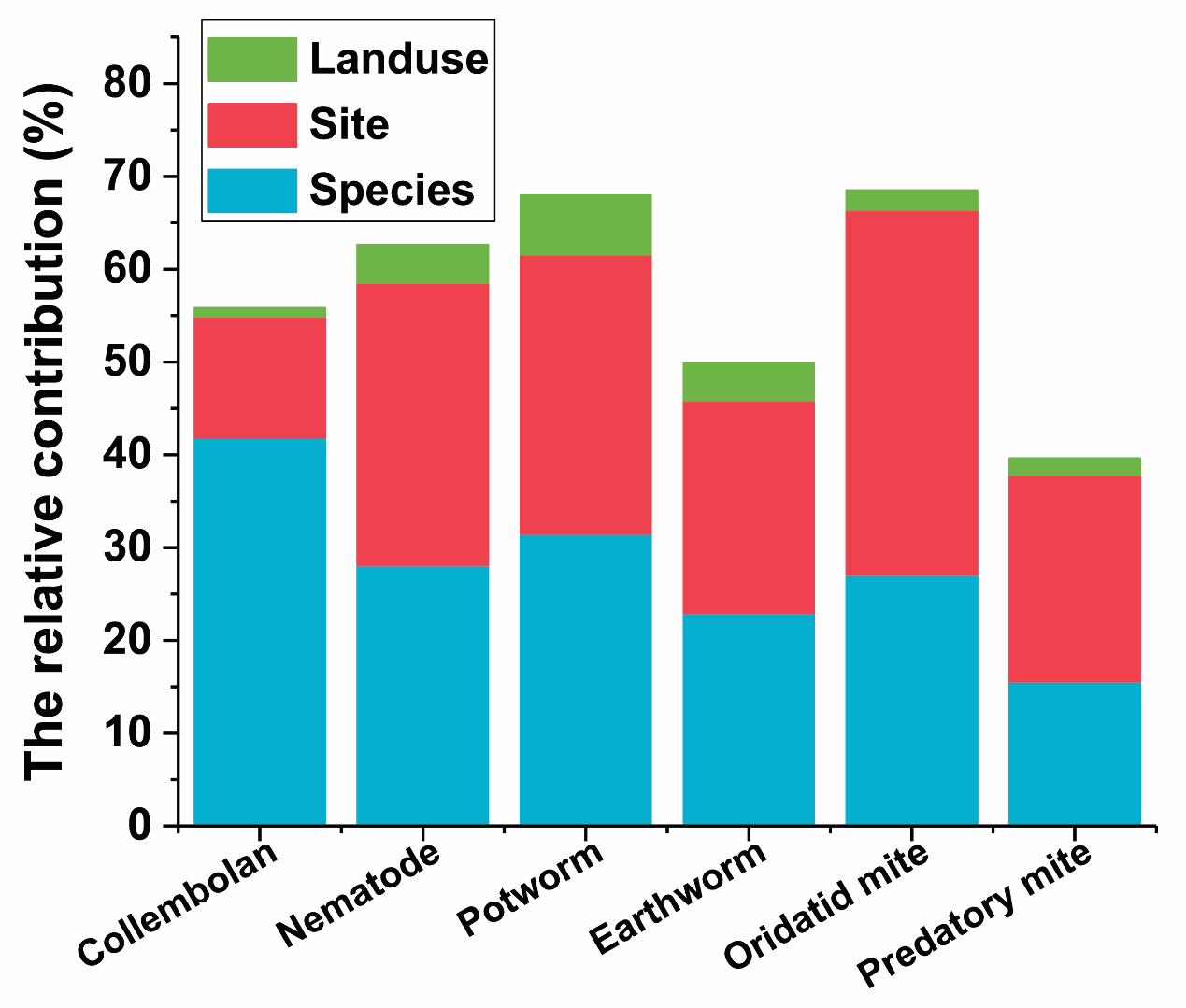


**Fig. S10.** The PERMANOVA analysis revealing the relative contribution of landuse, sampling site and soil faunal species to the variation of each soil faunal group microbiome.


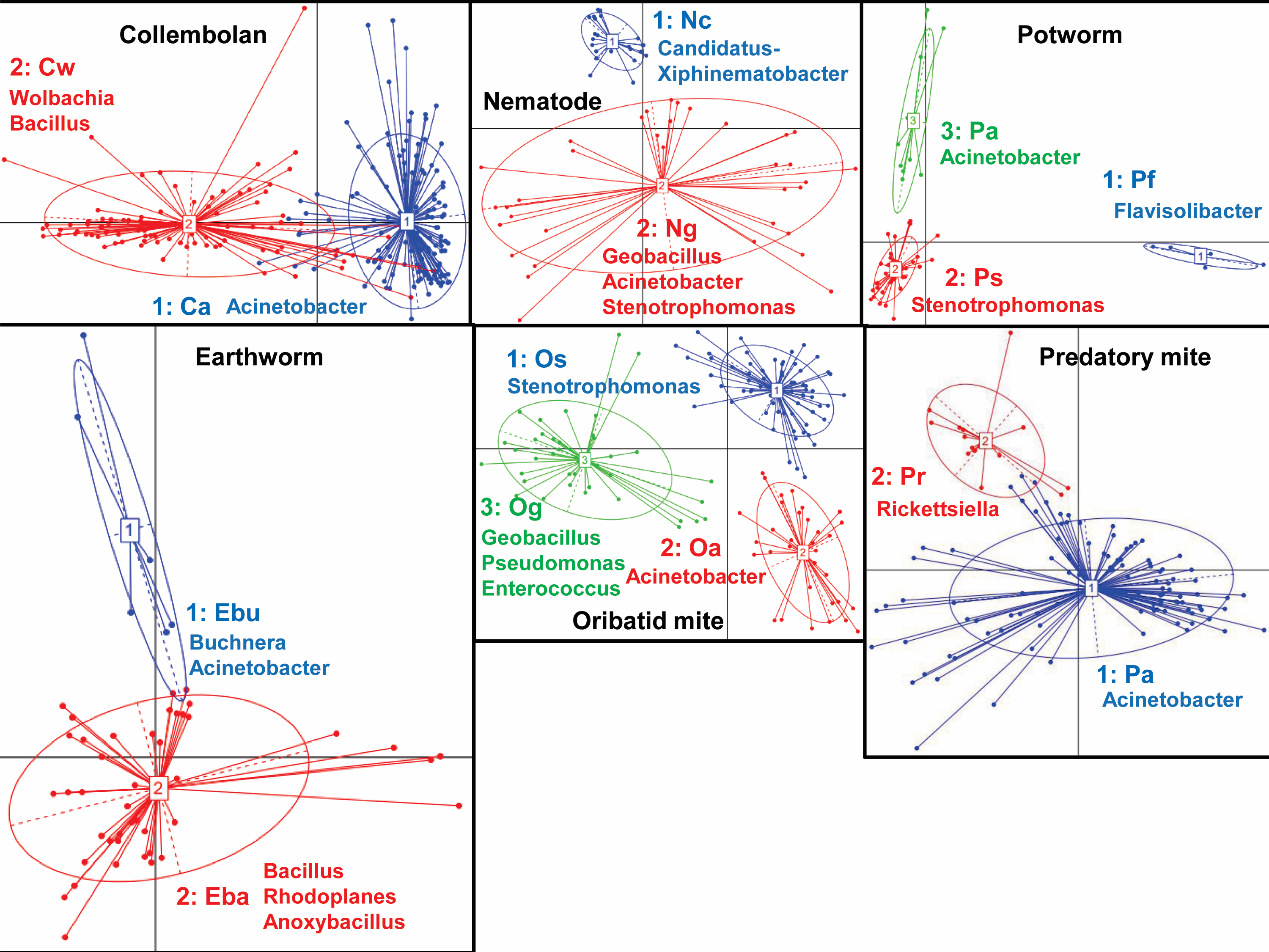


**Fig. S11.** **Enterotyping (clustering) of each soil faunal group, which was clustered using the Jensen–Shannon distance and partitioning around medoids method based on the relative abundance of bacterial genera.** Each point indicated a soil fauna sample. Representative bacterial genera of each enterotype were identified by the LDA Effect Size analysis. The collembolan, nematode, earthworm and predatory mite have two enterotypes (clusters), and the potworm and oribatid mite have three enterotypes (clusters), respectively.


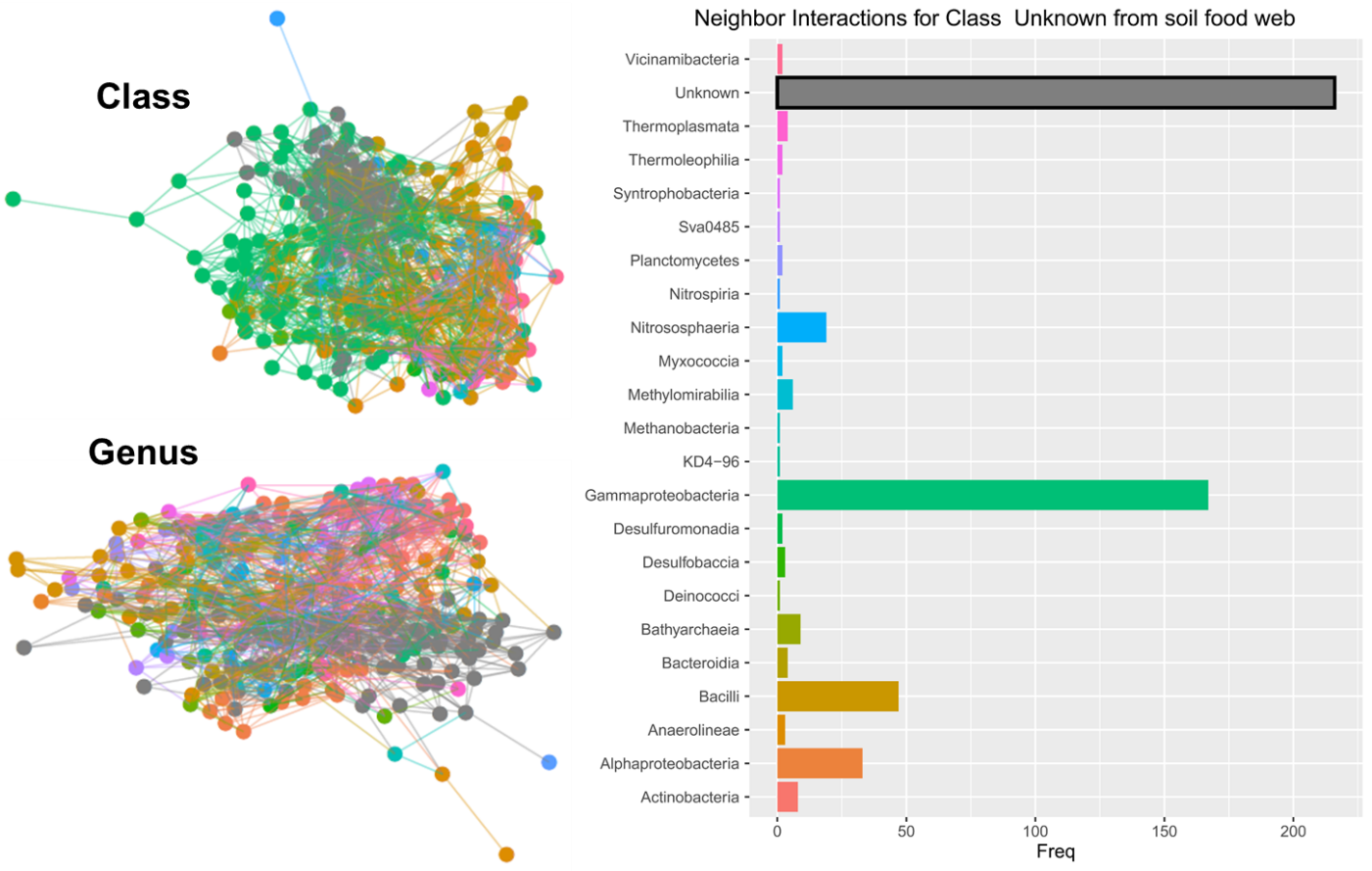


c

b

a

**Fig. S12.** Analysis of microbial network taxa interconnectedness from the soil food web microbiome. Microbial networks at the Class level (a) and Genus level (b). Nodes and edges are colored by class assignment, with gray nodes and edges representing Unknown taxa. (c) Bar graphs of the co-occurrence relationships of Unknown OTUs with other taxa at the class level within soil food web microbiome network. Y axis labels and colors signify the different classes with which Unknowns were found to co-occur. Unknown-Unknown relationships are represented in gray.


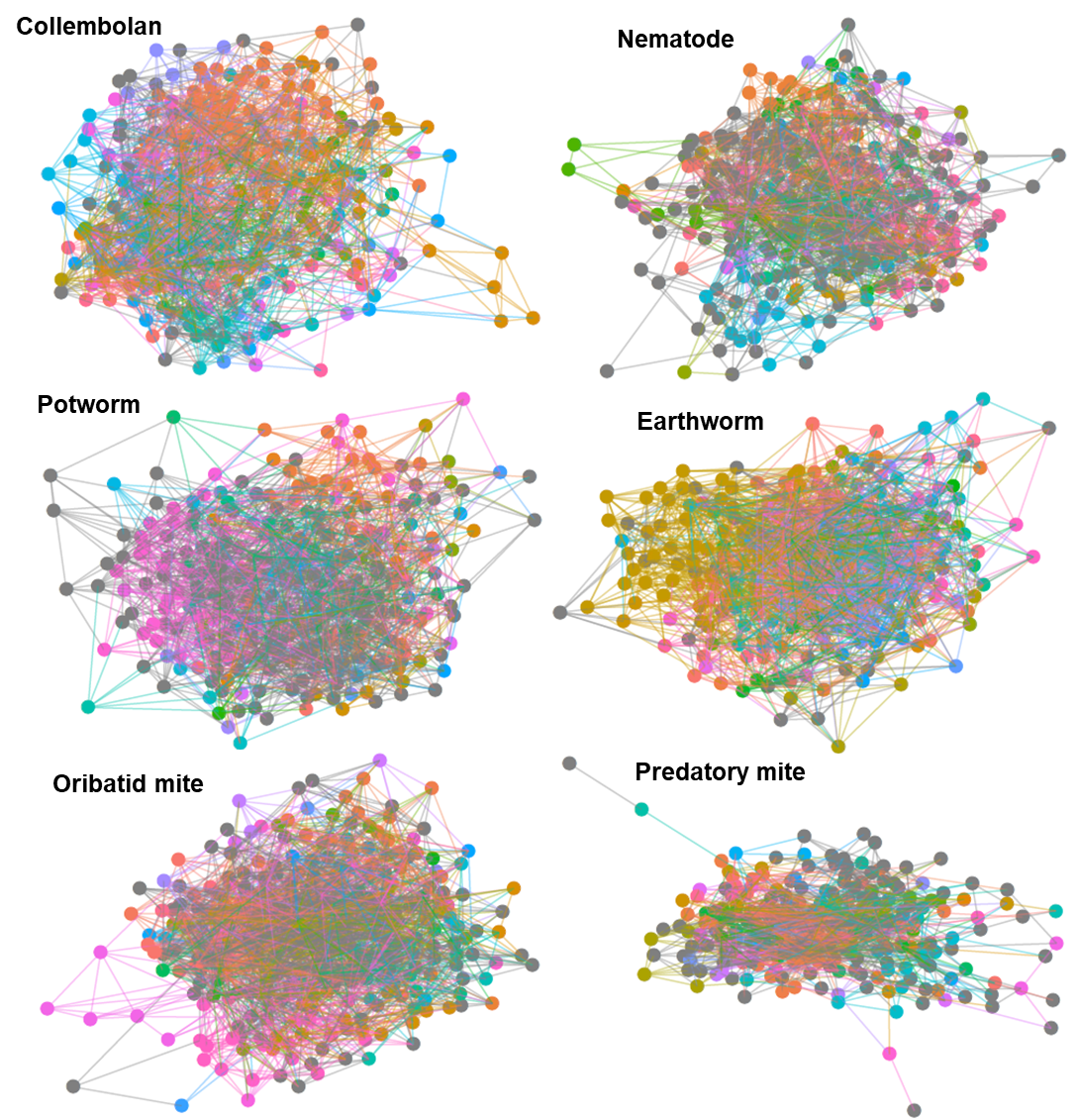


**Fig. S13.** Network analysis revealing microbial taxa interconnectedness at the Genus level. Nodes and edges are colored by class assignment, with gray nodes and edges representing Unknown taxa at the Genus level.


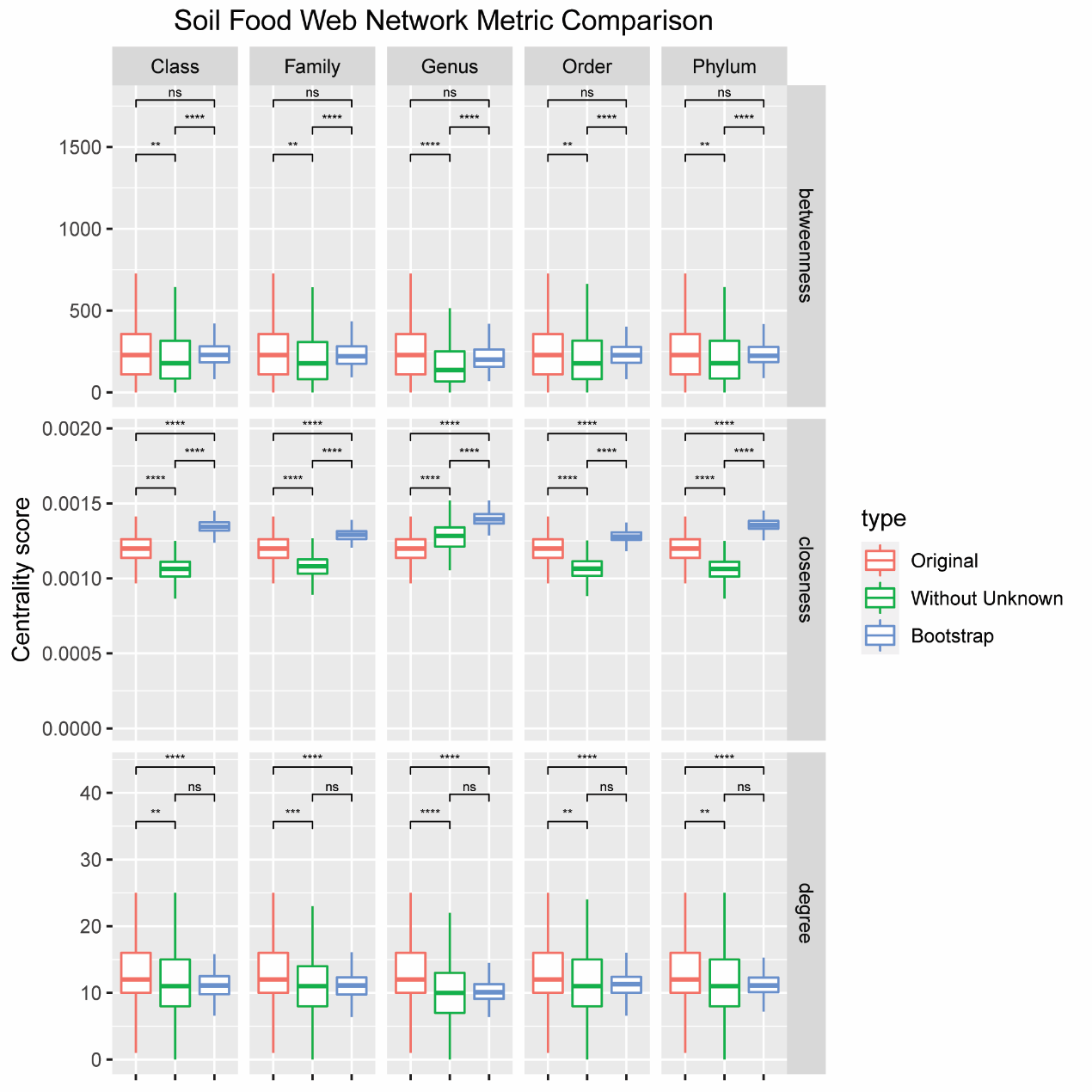


**Fig. S14.** Boxplots presenting the difference of betweenness, closeness, and degree centrality values of nodes between the three network types (Original-Without Unknown, Original-Bootstrap and Without Unknown-Bootstrap) at different taxonomic levels, which reflected effects of Unknown taxa on soil food web network metrics. The significance of result was assessed using the Wilcoxon pairwise comparison (significant level P < 0.05), and Holm adjusted P values were presented.


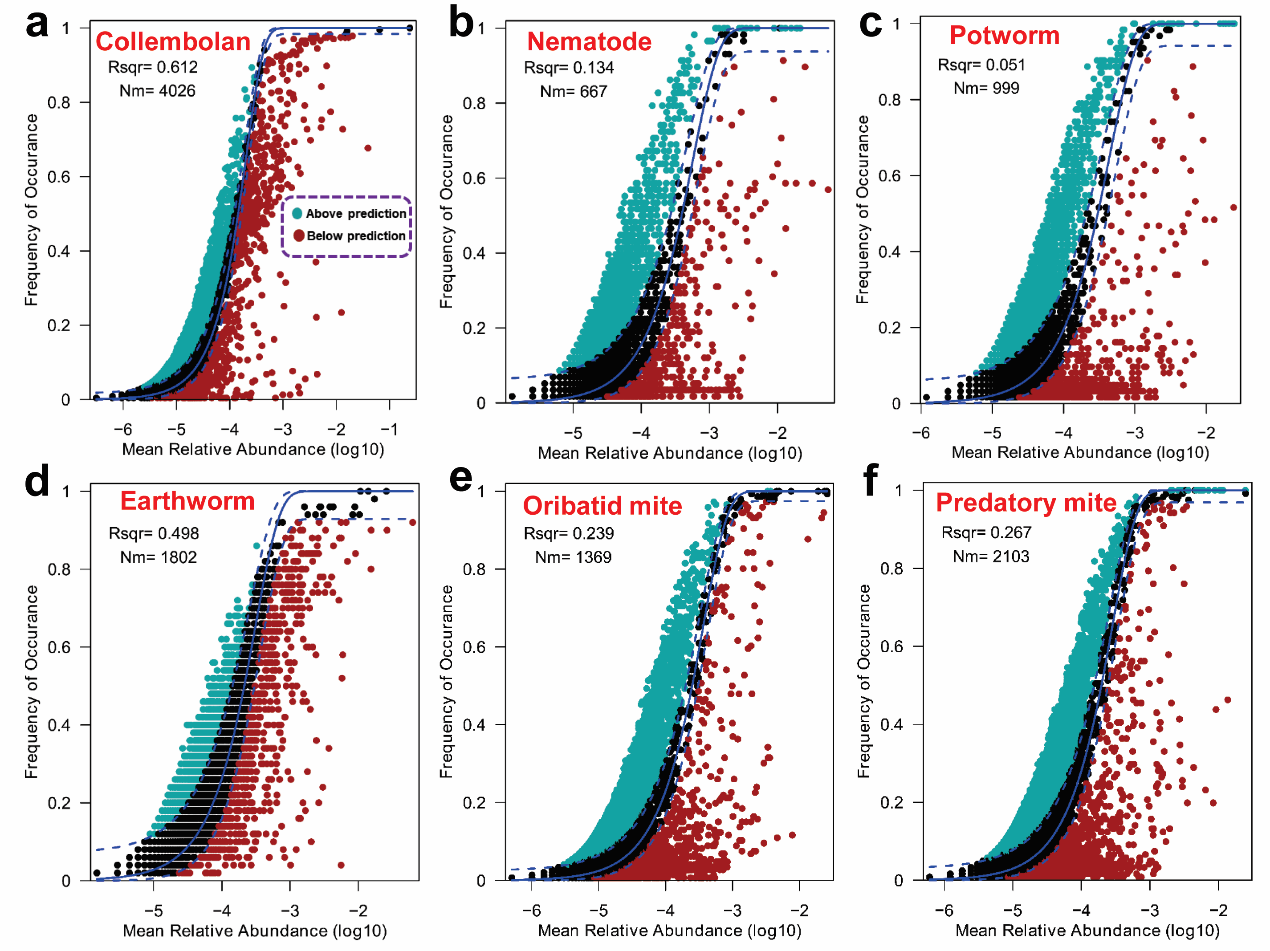


**Fig. S15.** Fit of neutral model for Collembolan (a), Nematode (b), Potworm (c), Earthworm (d), Oribatid mite (e) and predatory mite (g). The predicted occurrence frequency is shown as a solid black line and dashed blue lines indicate the 95% confidence interval around the neutral model prediction. Each point represents an OTU.


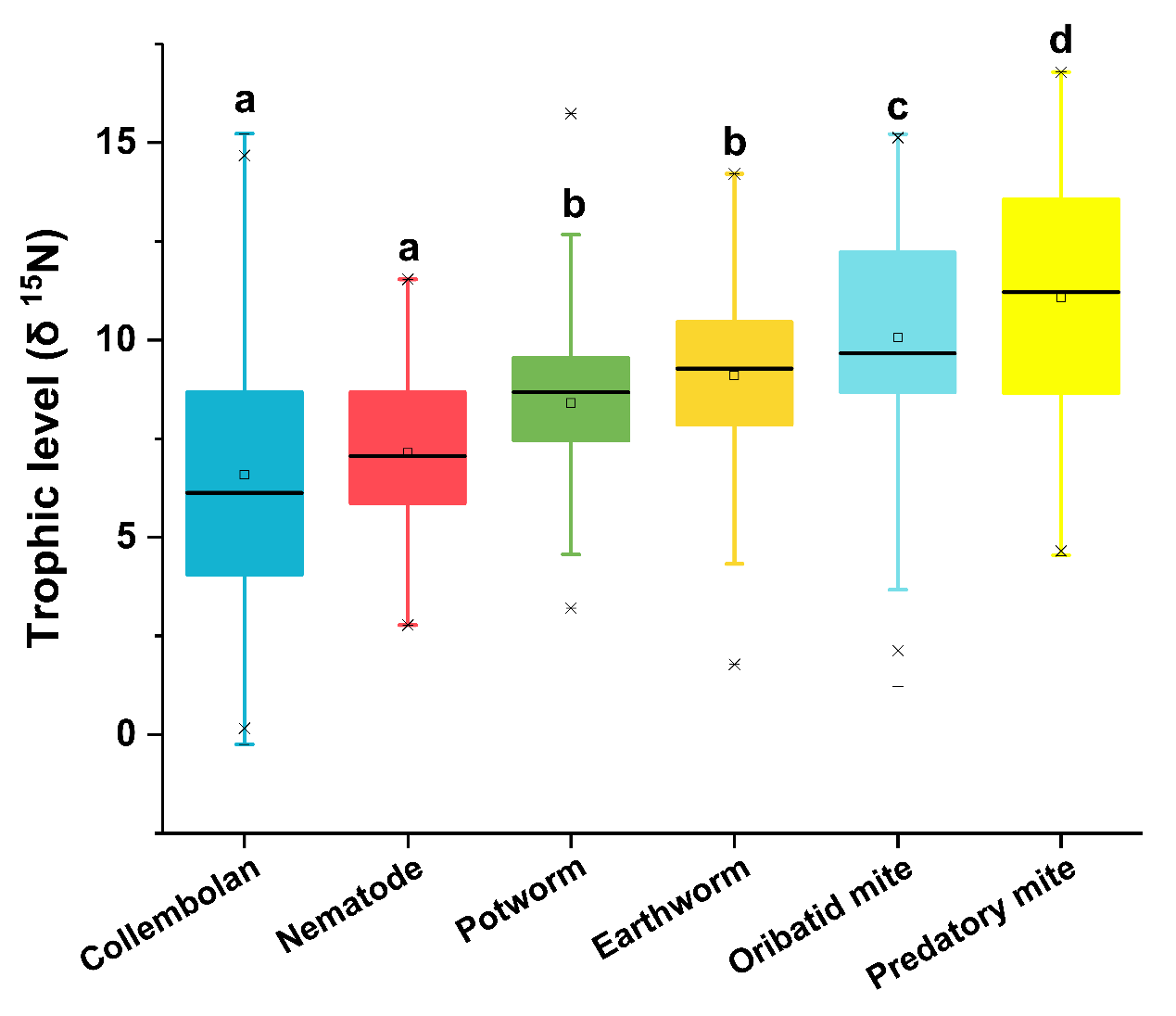


**Fig. S16.** Boxplot revealing natural ^15^N fractionation (δ ^15^N value) of different soil fauna (centre line, median; box limits, first and third quartiles; whiskers, 1.5 × interquartile range). Since all data conformed to a normal distribution, ANOVA with the Duncan test was used to compare the difference of δ ^15^N value between different animal tissues (Significance level: *P* = 0.05). The different letters indicate a significant difference between different animals. Natural ^15^N fractionation of animal body tissue (δ ^15^N value) is commonly used to represent the trophic level of animal in the ecosystem. To ensure the comparability of δ ^15^N value between different sites, we determined δ ^15^N value of litter at each site, and then obtained the adjusted δ ^15^N value by the measured δ ^15^N value of animal minus δ ^15^N value of litter. The adjusted δ ^15^N value was used in the Figure.


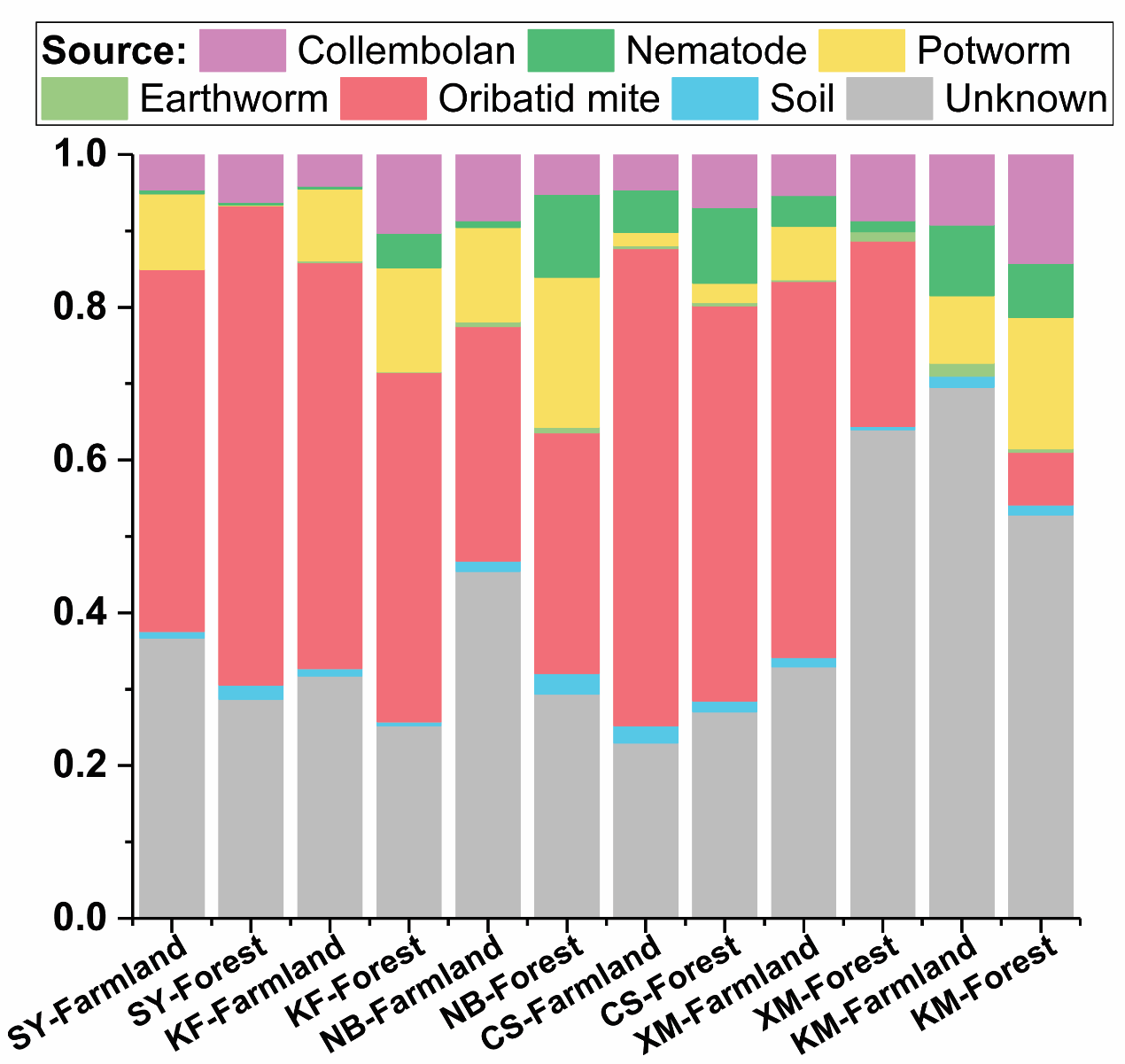


**Source proportion**

**Fig. S17.** FEAST estimations of source contribution to the sink (predatory mite) in each sampling site. The source (oribatid mite) was not included in the KM-Farmland, and the source (potworm) was not included in the XM-Forest.


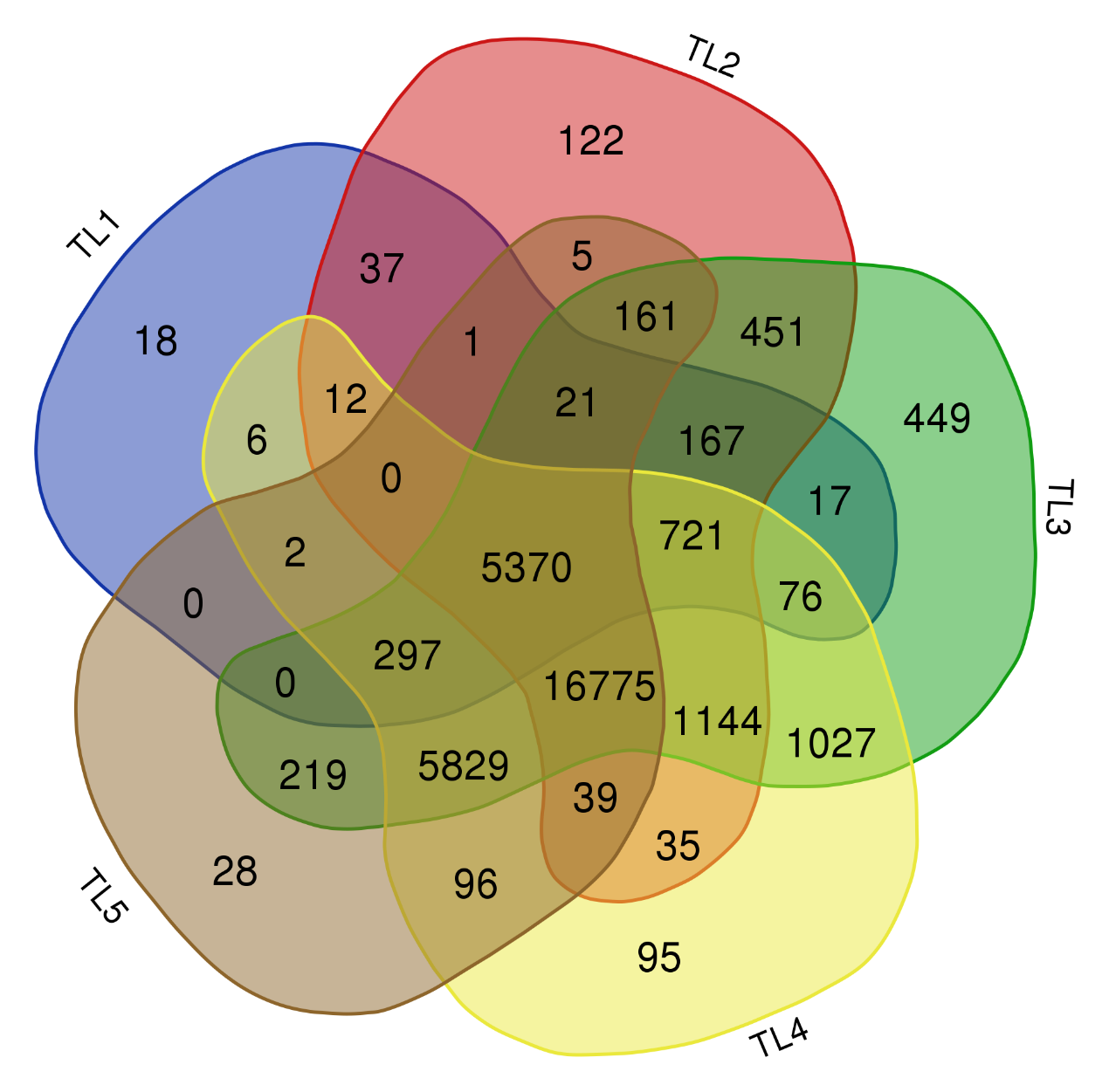


**Fig. S18.** Venn diagram revealing the shared OTUs number between different trophic level of soil fauna.


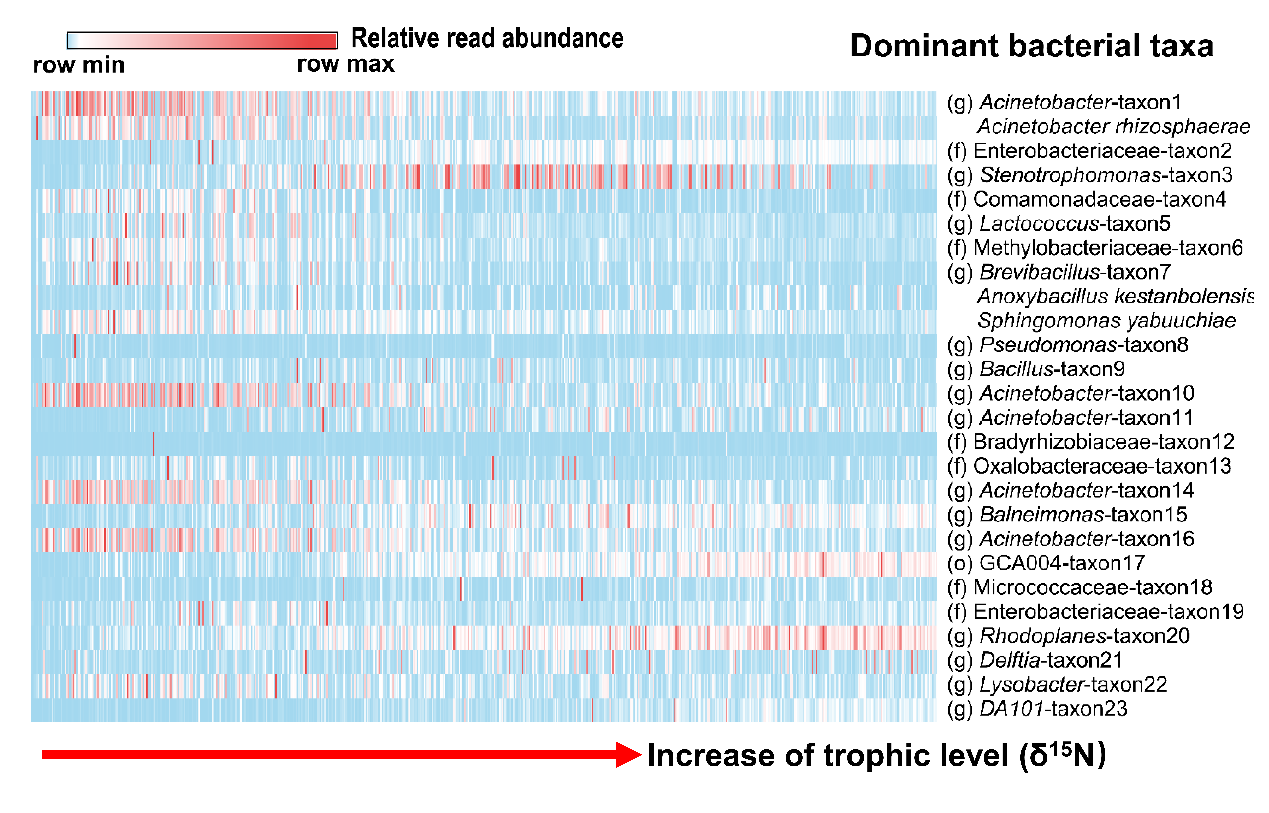


**Fig. S19.** Change in relative read abundance of 26 dominant bacterial taxa along an increase in trophic level (δ ^15^N value) of the soil fauna. Dominant bacterial taxa were defined by a relative read abundance > 0.1% and being found in more than 80% of soil fauna samples. The bacterial taxa were identified at the species level or higher possible taxonomic classification. The letters (o), (f) and (g) indicate the order, family and genus levels, respectively.


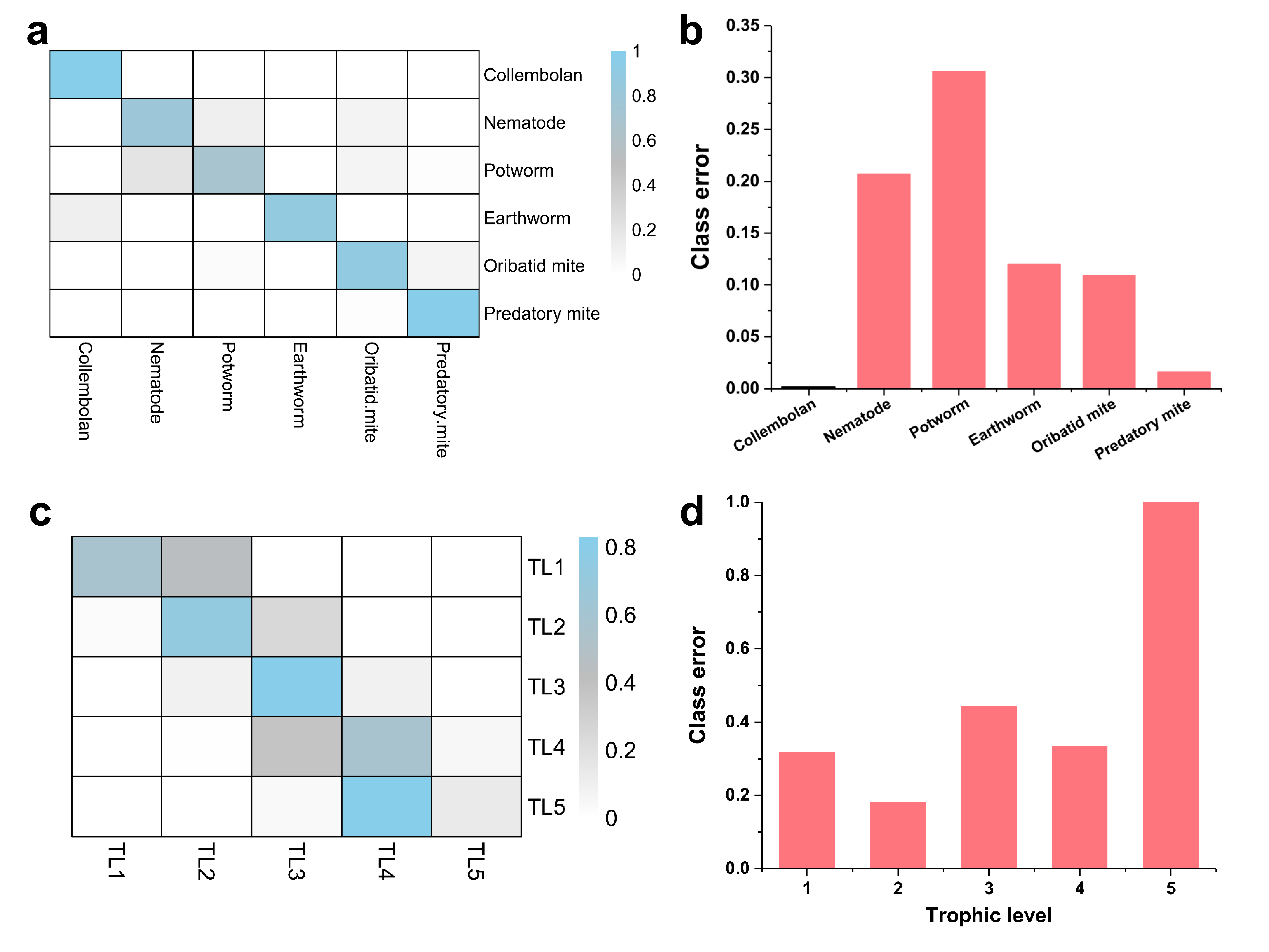


**Fig. S20.** Random forest classification of soil fauna microbial communities (including 58 bacterial taxa which were found in more than 70% of all soil fauna samples) based on different animal types (a and b) and trophic levels (c and d). a) and c) Each row of the confusion matrix from random forest analysis represent the soil fauna or trophic level, and the color intensity indicates within-group coherence and corresponds to the fraction of samples that were predicted by the classifier to belong to the soil fauna or trophic level specified by each column. b) and d) Class error estimates indicate the integrity of each soil fauna or trophic level given animal microbial communities analyzed. The “TL” indicates trophic level.


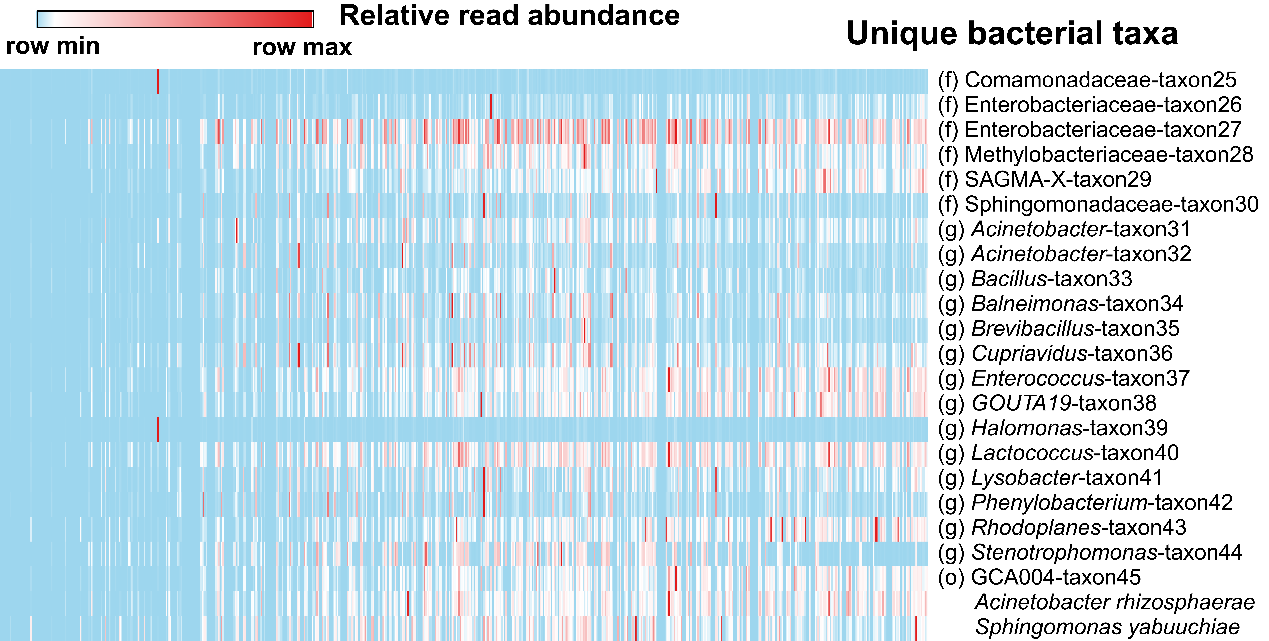


**(g) *Acinetobacter*_taxon46**

**(g) *Sphingomonas*_taxon47**


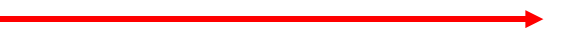
 **Increase in trophic level (δ^15^N)**

**Fig. S21.** Changes in relative read abundance of 23 unique bacterial taxa across increased trophic level as measured by δ ^15^N value (-0.25-16.79) in the soil fauna. Unique bacterial taxa were chosen on the basis of a maximal relative read abundance of > 1% in more than 50% of soil fauna samples. The bacterial taxa were presented at the OTU level and annotated with higher possible taxonomic classification. The (o), (f) and (g) indicate the order, family and genus levels, respectively.


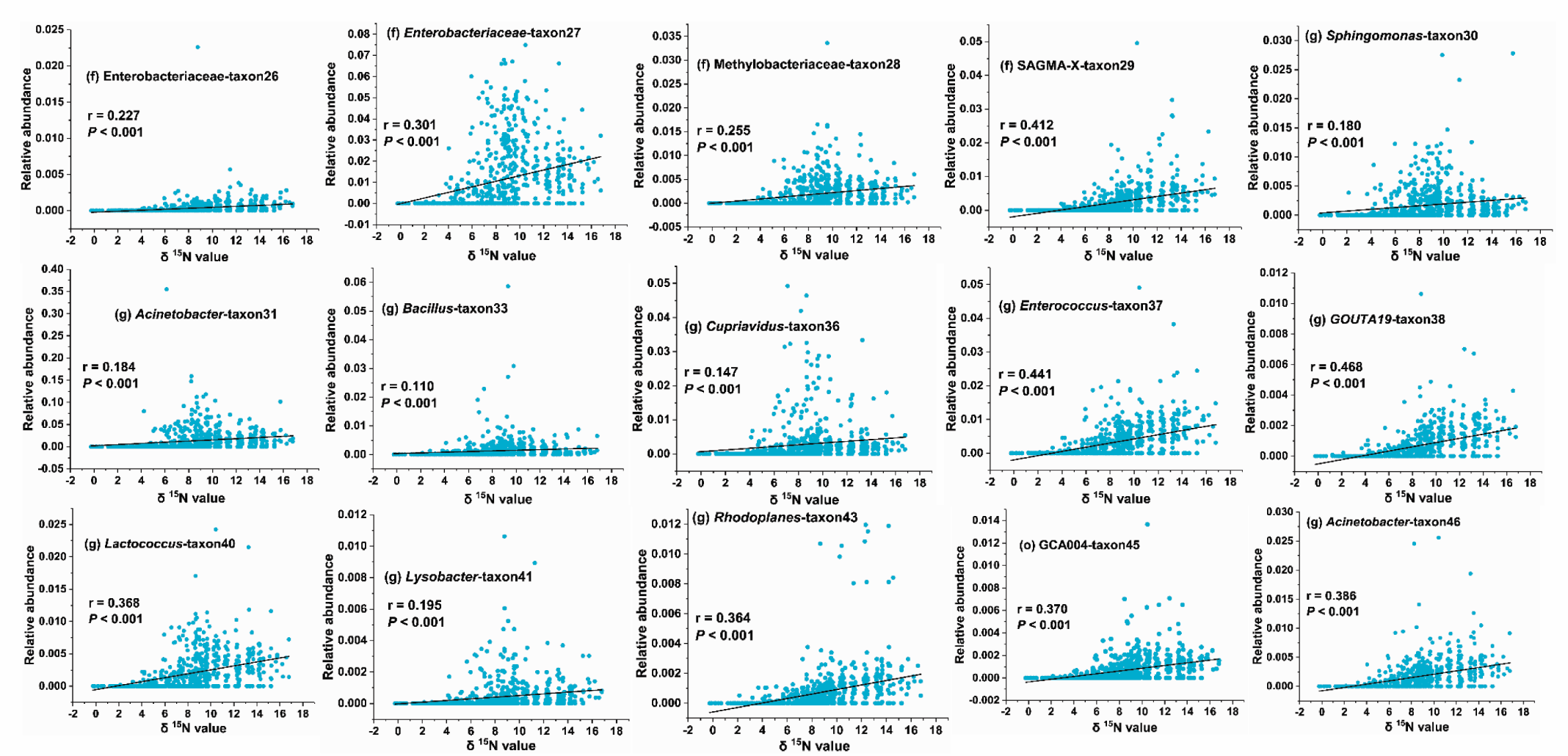


**Fig. S22.** Linear fitting diagrams revealing unique bacterial taxa that enriched with increasing δ ^15^N value (-0.25-16.79) in the soil fauna (*P* < 0.001).


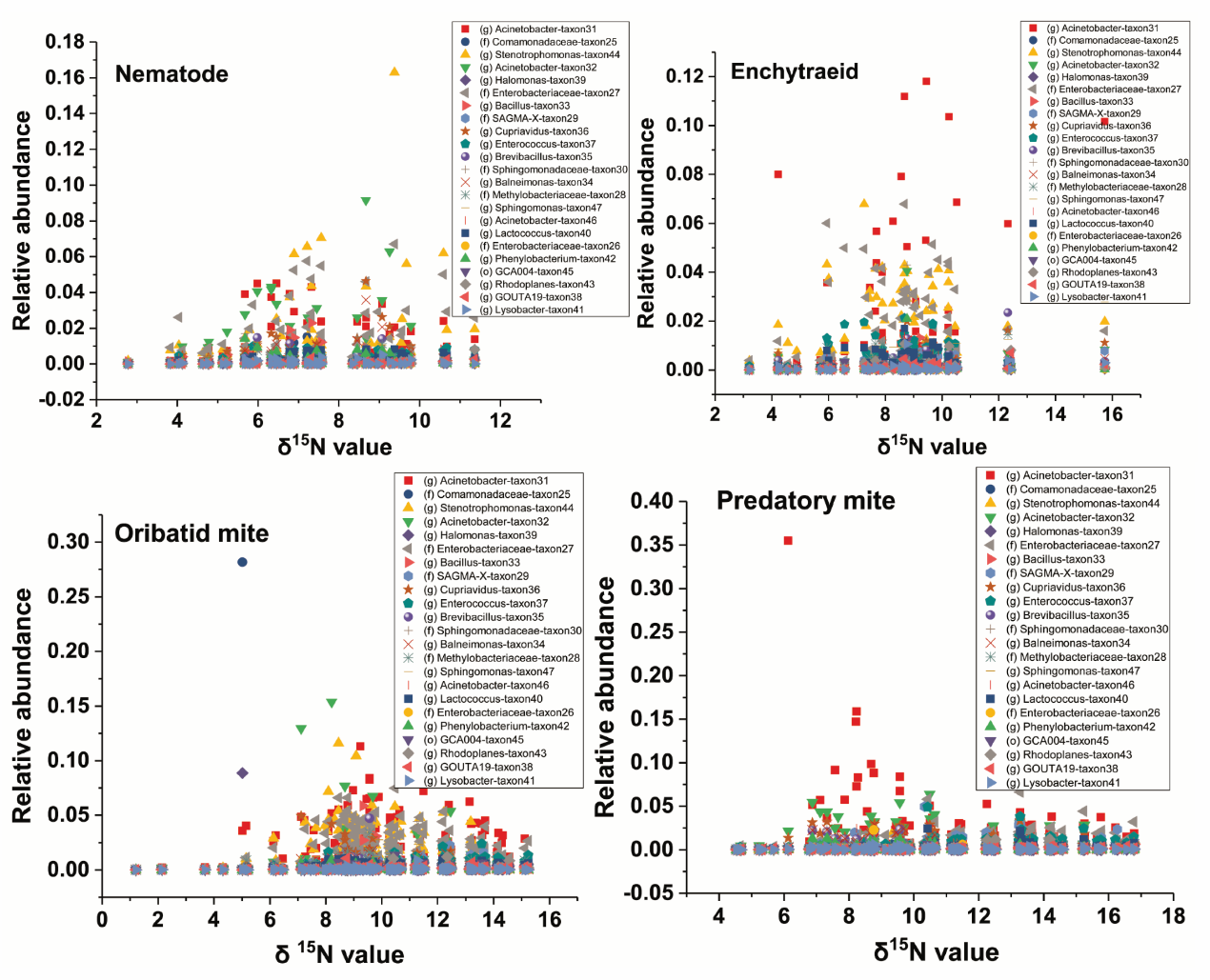


**Potworm**

**Fig. S23.** Scatter diagrams revealing changes in relative read abundance of 23 unique bacterial taxa across increased trophic level as measured by δ ^15^N value (-0.25-16.79) in the soil fauna. The 23 unique bacterial taxa all did not detected in collembolan and earthworm microbiomes. Therefore, diagrams of collembolan and earthworm did not been included. Unique bacterial taxa were chosen on the basis of a maximal relative read abundance of > 1% in more than 50% of soil fauna samples. The bacterial taxa were presented at the OTU level and annotated with higher possible taxonomic classification. The (o), (f) and (g) indicate the order, family and genus levels, respectively.


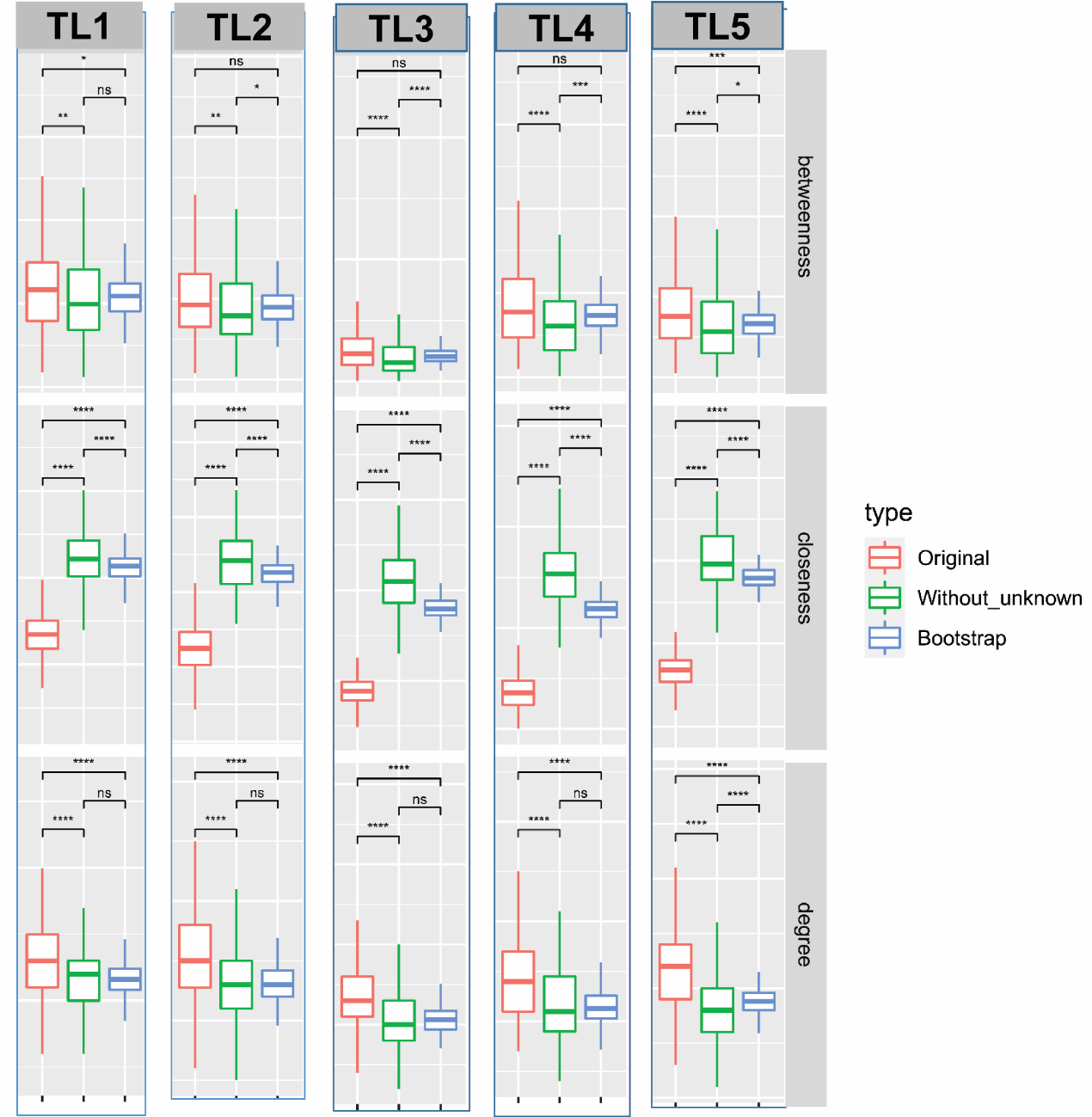


**Fig. S24.** Boxplots presenting the difference of betweenness, closeness, and degree centrality values of nodes between the three network types (Original-Without Unknown, Original-Bootstrap and Without Unknown-Bootstrap) at the Genus level, which reflected effects of Unknown taxa on different trophic level soil faunal network metrics. The significance of result was assessed using the Wilcoxon pairwise comparison (significant level P < 0.05), and Holm adjusted P values were presented. The “TL” indicated the trophic level of soil fauna based on natural ^15^N fractionation of animal body tissue. The “ns” indicated no significant difference.
