# Supplementary material for: Trophic level drives the host microbiome of soil invertebrates at a continental scale
Source: Microbiome. 2021 Sep 20;9:189. doi: 10.1186/s40168-021-01144-4 (PMC8454154; doi:10.1186/s40168-021-01144-4)
Supplement: Supplementary file 4 — Additional file 3. A diagram of the hierarchy based on the current knowledge of soil invertebrates revealing the relationship among collembola, nematode, potworm, earthworm, oribatid mite and predatory mite in the food web. [file 40168_2021_1144_MOESM4_ESM.pdf]

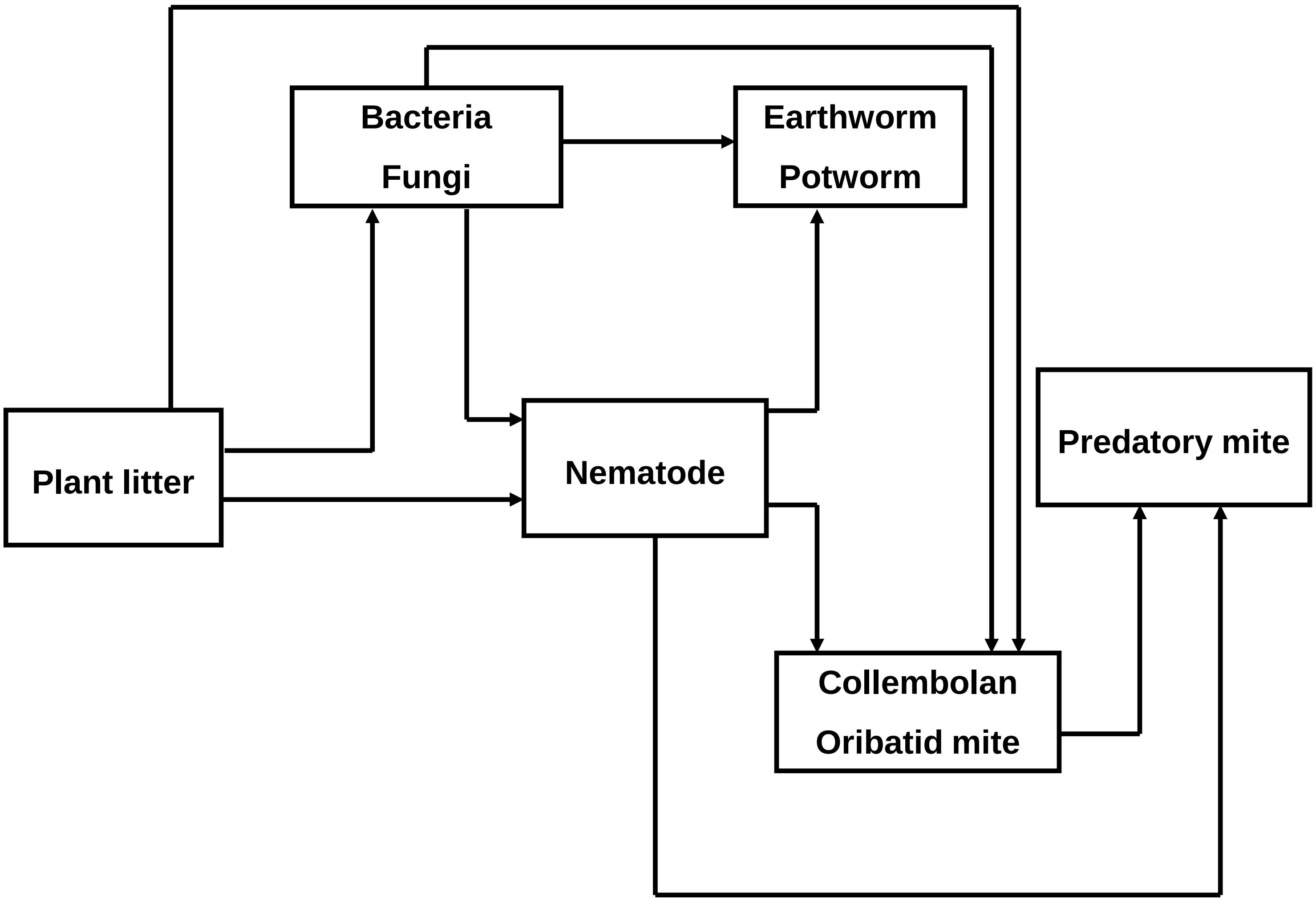

A diagram of the hierarchy based on the current knowledge of soil invertebrates revealing the relationship among collembolan, nematode, potworm, earthworm, oribatid mite and predatory mite in the food web.
