# Supplementary material for: Trophic level drives the host microbiome of soil invertebrates at a continental scale
Source: Microbiome. 2021 Sep 20;9:189. doi: 10.1186/s40168-021-01144-4 (PMC8454154; doi:10.1186/s40168-021-01144-4)
Supplement: Supplementary file 5 — Additional file 4. The R scripts. [file 40168_2021_1144_MOESM5_ESM.pdf]

Additive diversity partitioning

```
library(vegan)
y=read.csv(file.choose(),row.names=1)
y=t(y)
x=read.csv(file.choose(),row.names=1)
adipart(y, x, index=c("richness", "shannon", "simpson"),
        weights=c("unif", "prop"), relative = FALSE, nsimul=99,
        method = "r2dtable")
adipart(y~sample+landuse,data=x,index="shannon", nsimul=99, weights="unif")
```

Enterotyping

```
#Uncomment next two lines if R packages are already installed
#install.packages("cluster")
#install.packages("clusterSim")
library(cluster)
library(clusterSim)

#Download the example data and set the working directory
#setwd('<path_to_working_directory>')
data=read.table("collembolan1.txt", header=T, row.names=1, dec=".", sep="\t")
data=data[-1,]

dist.JSD <- function(inMatrix, pseudocount=0.000001, ...) {
  KLD <- function(x,y) sum(x *log(x/y))
  JSD<- function(x,y) sqrt(0.5 * KLD(x, (x+y)/2) + 0.5 * KLD(y, (x+y)/2))
  matrixColSize <- length(colnames(inMatrix))
  matrixRowSize <- length(rownames(inMatrix))
  colnames <- colnames(inMatrix)
  resultsMatrix <- matrix(0, matrixColSize, matrixColSize)

  inMatrix = apply(inMatrix,1:2,function(x) ifelse (x==0,pseudocount,x))

  for(i in 1:matrixColSize) {
    for(j in 1:matrixColSize) {
      resultsMatrix[i,j]=JSD(as.vector(inMatrix[,i]),
                             as.vector(inMatrix[,j]))
    }
  }
  colnames -> colnames(resultsMatrix) -> rownames(resultsMatrix)
  as.dist(resultsMatrix)->resultsMatrix
  attr(resultsMatrix, "method") <- "dist"
  return(resultsMatrix)
```

```
}

data.dist=dist.JSD(data)

pam.clustering=function(x,k) { # x is a distance matrix and k the number of clusters
   require(cluster)
   cluster = as.vector(pam(as.dist(x), k, diss=TRUE)$clustering)
   return(cluster)
}

data.cluster=pam.clustering(data.dist, k=3)

require(clusterSim)
nclusters = index.G1(t(data), data.cluster, d = data.dist, centrotypes = "medoids")

nclusters=NULL

for (k in 1:20) {
   if (k==1) {
      nclusters[k]=NA
   } else {
      data.cluster_temp=pam.clustering(data.dist, k)
      nclusters[k]=index.G1(t(data),data.cluster_temp,   d = data.dist,
                                          centrotypes = "medoids")
   }
}

plot(nclusters, type="h", xlab="k clusters", ylab="CH index",main="Optimal number of clusters")

obs.silhouette=mean(silhouette(data.cluster, data.dist)[,3])
cat(obs.silhouette) #0.1899451


# noise.removal <- function(dataframe, percent=0.01, top=NULL){
dataframe->Matrix
bigones <- rowSums(Matrix)*100/(sum(rowSums(Matrix))) > percent
Matrix_1 <- Matrix[bigones,]
print(percent)
return(Matrix_1)}
#data=noise.removal(data, percent=0.01)

library(ade4)
data.cluster1=pam.clustering(data.dist, k=3)
```

```
data.cluster=pam.clustering(data.dist, k=2)
## plot 1
obs.pca=dudi.pca(data.frame(t(data)), scannf=F, nf=10)
obs.bet=bca(obs.pca, fac=as.factor(data.cluster1), scannf=F, nf=k-1)
dev.new()
s.class(obs.bet$ls, fac=as.factor(data.cluster), grid=F,sub="Between-class analysis", col=c(4,2,3))
s.class(obs.bet$ls, fac=as.factor(data.cluster), grid=F, cell=0, cstar=0, col=c(4,2,3))
?s.class
obs.bet$ls
data.cluster
fac=as.factor(data.cluster)
fac


#plot 2
obs.pcoa=dudi.pco(data.dist, scannf=F, nf=3)
dev.new()
s.class(obs.pcoa$li, fac=as.factor(data.cluster), grid=F,sub="Principal coordiante analysis")


require(devtools)
install_github("tapj/BiotypeR")
```

PERMANOVA (Adonis test)

```
library(vegan)
otu <- read.delim('family-args.txt', row.names = 1, sep = '\t', stringsAsFactors = FALSE, check.names = FALSE)
otu <- data.frame(t(otu))
group <- read.delim('sfw-group.txt', sep = '\t', stringsAsFactors = FALSE)
adonis_result <- adonis(otu~site, group, distance = 'bray', permutations = 999)
adonis_result <- adonis(dis1~landuse, group, permutations = 999)
dis <- read.delim('oribatid-distance.txt', row.names = 1, sep = '\t', stringsAsFactors = FALSE, check.names = FALSE)
dis1 <- as.dist(dis)
adonis_result <- adonis(dis1~species, group, permutations = 999)
adonis_result
```

Neutral community model

```
##Fits the neutral model from Sloan et al. 2006 to an OTU table and returns several fitting statistics.
##Alternatively, will return predicted occurrence frequencies for each OTU based on their
```

abundance in the metacommunity

```
##Install the following packages if they haven't been availabled in your computer yet.
library(Hmisc)
library(minpack.lm)
library(stats4)
#using Non-linear least squares (NLS) to calculate R2:
#spp: A community table with taxa as rows and samples as columns
spp<-read.csv(file.choose(),head=T,stringsAsFactors=F,row.names=1,sep = "\t")
spp<-t(spp)


setwd("C:/Users/Dong Zhu/Desktop/")
spp <- read.delim('feature-table-norm.txt', row.names = 1, sep = '\t', stringsAsFactors = FALSE, check.names = FALSE)
spp <- data.frame(t(spp))


N <- mean(apply(spp, 1, sum))
p.m <- apply(spp, 2, mean)
p.m <- p.m[p.m != 0]
p <- p.m/N
spp.bi <- 1*(spp>0)
freq <- apply(spp.bi, 2, mean)
freq <- freq[freq != 0]
C <- merge(p, freq, by=0)
C <- C[order(C[,2]),]
C <- as.data.frame(C)
C.0 <- C[!(apply(C, 1, function(y) any(y == 0))),]
p <- C.0[,2]
freq <- C.0[,3]
names(p) <- C.0[,1]
names(freq) <- C.0[,1]
d = 1/N
##Fit model parameter m (or Nm) using Non-linear least squares (NLS)
m.fit <- nlsLM(freq ~ pbeta(d, N*m*p, N*m*(1 -p), lower.tail=FALSE),start=list(m=0.1))
m.fit #get the m value
m.ci <- confint(m.fit, 'm', level=0.95)


##Fit neutral model parameter m (or Nm) using Maximum likelihood estimation (MLE)
sncm.LL <- function(m, sigma){
   R = freq - pbeta(d, N*m*p, N*m*(1-p), lower.tail=FALSE)
   R = dnorm(R, 0, sigma)
   -sum(log(R))
```

```
}
m.mle <- mle(sncm.LL, start=list(m=0.1, sigma=0.1), nobs=length(p))

##Calculate Akaike's Information Criterion (AIC)
aic.fit <- AIC(m.mle, k=2)
bic.fit <- BIC(m.mle)
aic.fit

##Calculate AIC for binomial model
bino.LL <- function(mu, sigma){
  R = freq - pbinom(d, N, p, lower.tail=FALSE)
  R = dnorm(R, mu, sigma)
  -sum(log(R))
}
bino.mle <- mle(bino.LL, start=list(mu=0, sigma=0.1), nobs=length(p))

aic.bino <- AIC(bino.mle, k=2)
bic.bino <- BIC(bino.mle)
aic.bino

freq.pred <- pbeta(d, N*coef(m.fit)*p, N*coef(m.fit)*(1 -p), lower.tail=FALSE)
pred.ci <- binconf(freq.pred*nrow(spp), nrow(spp), alpha=0.05, method="wilson", return.df=TRUE)
Rsqr <- 1 - (sum((freq - freq.pred)^2))/(sum((freq - mean(freq))^2))
Rsqr# get the R2 value

#Optional: write 3 files: p.csv, freq.csv and freq.pred.csv
write.csv(p, file = "C:/Users/Dong Zhu/Desktop/p.csv")
write.csv(freq, file = "C:/Users/Dong Zhu/Desktop/freq.csv")
write.csv(freq.pred, file = "C:/Users/Dong Zhu/Desktop/freq.pred.csv")


#Drawing the figure using grid package:
#p is the mean relative abundance
#freq is occurrence frequency
#freq.pred is predicted occurrence frequency
bacnlsALL <-data.frame(p,freq,freq.pred,pred.ci[,2:3])
write.csv(bacnlsALL, file = "C:/Users/Dong Zhu/Desktop/bacnlsALL.csv")
inter.col<-rep('black',nrow(bacnlsALL))
inter.col[bacnlsALL$freq <= bacnlsALL$Lower]<-'#A52A2A'#define the color of below points
inter.col[bacnlsALL$freq >= bacnlsALL$Upper]<-'#29A6A6'#define the color of up points
library(grid)
grid.newpage()
pushViewport(viewport(h=0.6,w=0.6))
```

```
pushViewport(dataViewport(xData=range(log10(bacnlsALL$p)),
yData=c(0,1.02),extension=c(0.02,0)))
grid.rect()
grid.points(log10(bacnlsALL$p), bacnlsALL$freq,pch=20,gp=gpar(col=inter.col,cex=0.7))
grid.yaxis()
grid.xaxis()
grid.lines(log10(bacnlsALL$p),bacnlsALL$freq.pred,gp=gpar(col='blue',lwd=2),default='native')


grid.lines(log10(bacnlsALL$p),bacnlsALL$Lower ,gp=gpar(col='blue',lwd=2,lty=2),default='native')
grid.lines(log10(bacnlsALL$p),bacnlsALL$Upper,gp=gpar(col='blue',lwd=2,lty=2),default='native')
grid.text(y=unit(0,'npc')-unit(2.5,'lines'),label='Mean Relative Abundance (log10)', gp=gpar(fontface=2))
grid.text(x=unit(0,'npc')-unit(3,'lines'),label='Frequency of Occurance',gp=gpar(fontface=2),rot=90)
#grid.text(x=unit(0,'npc')-unit(-1,'lines'), y=unit(0,'npc')-unit(-15,'lines'),label='Mean Relative Abundance (log)', gp=gpar(fontface=2))
#grid.text(round(coef(m.fit)*N),x=unit(0,'npc')-unit(-5,'lines'), y=unit(0,'npc')-unit(-15,'lines'),gp=gpar(fontface=2))
#grid.text(label = "Nm=",x=unit(0,'npc')-unit(-3,'lines'), y=unit(0,'npc')-unit(-15,'lines'),gp=gpar(fontface=2))
#grid.text(round(Rsqr,2),x=unit(0,'npc')-unit(-5,'lines'), y=unit(0,'npc')-unit(-16,'lines'),gp=gpar(fontface=2))
#grid.text(label = "Rsqr=",x=unit(0,'npc')-unit(-3,'lines'), y=unit(0,'npc')-unit(-16,'lines'),gp=gpar(fontface=2))
draw.text <- function(just, i, j) {
grid.text(paste("Rsqr=",round(Rsqr,3),"\n","Nm=",round(coef(m.fit)*N)), x=x[j], y=y[i], just=just)
#grid.text(deparse(substitute(just)), x=x[j], y=y[i] + unit(2, "lines"),
#          gp=gpar(col="grey", fontsize=8))
  }
x <- unit(1:4/5, "npc")
y <- unit(1:4/5, "npc")
draw.text(c("centre", "bottom"), 4, 1)
```

Phylogenetic bin based null model analysis (iCAMP)

```
rm(list=ls())
t0=Sys.time()
wd="/home/dongzhu/SFW2"
com.file="SFWotus.txt"
```

```
tree.file="tree.nwk"
treat.file="tre.txt"
env.file="env.txt"
save.wd="/home/dongzhu/SFW2/out"
if(!dir.exists(save.wd)){dir.create(save.wd)}
prefix="Test"
rand.time=100
nworker=40
memory.G=900
library(iCAMP)
library(ape)
setwd(wd)
comm=t(read.table(com.file, header = TRUE, sep = "\t", row.names = 1,
                  as.is = TRUE, stringsAsFactors = FALSE, comment.char = "",
                  check.names = FALSE))
tree=read.tree(file = tree.file)
treat=read.table(treat.file, header = TRUE, sep = "\t", row.names = 1,
                 as.is = TRUE, stringsAsFactors = FALSE, comment.char = "",
                 check.names = FALSE)

env=read.table(env.file, header = TRUE, sep = "\t", row.names = 1,
               as.is = TRUE, stringsAsFactors = FALSE, comment.char = "",
               check.names = FALSE)
sampid.check=match.name(rn.list=list(comm=comm,treat=treat,env=env))
treat=sampid.check$treat
comm=sampid.check$comm
comm=comm[,colSums(comm)>0,drop=FALSE] # if some unmatched samples were removed, some OTUs may become ghosts, then you may use this line to remove them if necessary.
env=sampid.check$env # skip this if you do not have env.file
spid.check=match.name(cn.list=list(comm=comm),tree.list=list(tree=tree))
comm=spid.check$comm
tree=spid.check$tree
setwd(save.wd)
if(!file.exists("pd.desc"))
{
  pd.big=iCAMP::pdist.big(tree = tree, wd=save.wd, nworker = nworker, memory.G = memory.G)
  # output files:
  # path.rda: a R object to list all the nodes and  edge lengthes from root to every tip. saved in R data format. an intermediate output when claculating phylogenetic distance matrix.
  # pd.bin: BIN file (backingfile) generated by function big.matrix in R package bigmemory. This is the big matrix storing pairwise phylogenetic distance values. By using this bigmemory format file, we will not need memory but hard disk when calling big matrix for calculation.
```

```
  # pd.desc: the DESC file (descriptorfile) to hold the backingfile (pd.bin) description.
  # pd.taxon.name.csv: comma delimited csv file storing the IDs of tree tips (OTUs), serving
as the row/column names of the big phylogenetic distance matrix.
}else{
  # if you already calculated the phylogenetic distance matrix in a previous run
  pd.big=list()
  pd.big$tip.label=read.csv(paste0(save.wd,"/pd.taxon.name.csv"),row.names =
1,stringsAsFactors = FALSE)[,1]
  pd.big$pd.wd=save.wd
  pd.big$pd.file="pd.desc"
  pd.big$pd.name.file="pd.taxon.name.csv"
}
setwd(save.wd)
niche.dif=iCAMP::dniche(env = env,comm = comm,method = "niche.value",
                        nworker = nworker,out.dist=FALSE,bigmemo=TRUE,
                        nd.wd=save.wd)
ds = 0.2
bin.size.limit = 96
phylobin=taxa.binphy.big(tree = tree, pd.desc = pd.big$pd.file,pd.spname = pd.big$tip.label,
                         pd.wd = pd.big$pd.wd, ds = ds, bin.size.limit = bin.size.limit,
                         nworker = nworker)
sp.bin=phylobin$sp.bin[,3,drop=FALSE]
sp.ra=colMeans(comm/rowSums(comm))
abcut=3 # you may remove some species, if they are too rare to perform reliable correlation
test.
commc=comm[,colSums(comm)>=abcut,drop=FALSE]
dim(commc)
spname.use=colnames(commc)
binps=iCAMP::ps.bin(sp.bin = sp.bin,sp.ra = sp.ra,spname.use = spname.use,
                    pd.desc = pd.big$pd.file, pd.spname = pd.big$tip.label, pd.wd =
pd.big$pd.wd,
                    nd.list = niche.dif$nd,nd.spname = niche.dif$names,ndbig.wd =
niche.dif$nd.wd,
                    cor.method = "pearson",r.cut = 0.1, p.cut = 0.05, min.spn = 5)
if(file.exists(paste0(prefix,".PhyloSignalSummary.csv"))){appendy=TRUE;col.namesy=FALSE}el
se{appendy=FALSE;col.namesy=TRUE}
write.table(data.frame(ds=ds,n.min=bin.size.limit,binps$Index),file =
paste0(prefix,".PhyloSignalSummary.csv"),
            append = appendy, quote=FALSE, sep=",", row.names = FALSE,col.names =
col.namesy)
if(file.exists(paste0(prefix,".PhyloSignalDetail.csv"))){appendy2=TRUE;col.namesy2=FALSE}els
e{appendy2=FALSE;col.namesy2=TRUE}
write.table(data.frame(ds=ds,n.min=bin.size.limit,binID=rownames(binps$detail),binps$detai
l),file = paste0(prefix,".PhyloSignalDetail.csv"),
```

```
                    append = appendy2, quote = FALSE, sep = ",", row.names = FALSE, col.names = col.namesy2)
bin.size.limit = 96 # For real data, usually use a proper number according to phylogenetic signal test or try some settings then choose the reasonable stochasticity level. our experience is 12, or 24, or 48. but for this example dataset which is too small, have to use 5.
sig.index="Confidence" # see other options in help document of icamp.big.
icres=iCAMP::icamp.big(comm=comm, pd.desc = pd.big$pd.file, pd.spname=pd.big$tip.label,
                       pd.wd = pd.big$pd.wd, rand = rand.time, tree=tree,
                       prefix = prefix, ds = 0.2, pd.cut = NA, sp.check = TRUE,
                       phylo.rand.scale = "within.bin", taxa.rand.scale = "across.all",
                       phylo.metric = "bMPD", sig.index=sig.index, bin.size.limit = bin.size.limit,
                       nworker = nworker, memory.G = memory.G, rtree.save = FALSE, detail.save = TRUE,
                       qp.save = FALSE, detail.null = FALSE, ignore.zero = TRUE, output.wd = save.wd,
                       correct.special = TRUE, unit.sum = rowSums(comm), special.method = "depend",
                       ses.cut = 1.96, rc.cut = 0.95, conf.cut=0.975, omit.option = "no",meta.ab = NULL)
icbin=icamp.bins(icamp.detail = icres$detail,treat = treat,
                 clas=NULL,silent=FALSE, boot = TRUE,
                 rand.time = rand.time,between.group = TRUE)
save(icbin,file = paste0(prefix,".iCAMP.Summary.rda")) # just to archive the result. rda file is automatically compressed, and easy to load into R.
write.csv(icbin$Pt,file = paste0(prefix,".ProcessImportance_EachGroup.csv"),row.names = FALSE)
write.csv(icbin$Ptk,file = paste0(prefix,".ProcessImportance_EachBin_EachGroup.csv"),row.names = FALSE)
write.csv(icbin$Ptuv,file = paste0(prefix,".ProcessImportance_EachTurnover.csv"),row.names = FALSE)
write.csv(icbin$BPtk,file = paste0(prefix,".BinContributeToProcess_EachGroup.csv"),row.names = FALSE)
i=1
treat.use=treat[,i,drop=FALSE]
icamp.result=icres$CbMPDiCBraya
icboot=iCAMP::icamp.boot(icamp.result = icamp.result,treat = treat.use,rand.time = rand.time,
                         compare = TRUE,silent = FALSE,between.group = TRUE,ST.estimation = TRUE)
save(icboot,file=paste0(prefix,".iCAMP.Boot.",colnames(treat)[i],".rda"))
write.csv(icboot$summary,file = paste0(prefix,".iCAMP.BootSummary.",colnames(treat)[i],".csv"),row.names = FALSE)
```

```
write.csv(icboot$compare,file =
paste0(prefix,".iCAMP.Compare.",colnames(treat)[i],".csv"),row.names = FALSE)
i=2
treat.use=treat[,i,drop=FALSE]
icamp.result=icres$CbMPDiCBraya
icboot=iCAMP::icamp.boot(icamp.result = icamp.result,treat = treat.use,rand.time =
rand.time,
                         compare = TRUE,silent = FALSE,between.group =
TRUE,ST.estimation = TRUE)
save(icboot,file=paste0(prefix,".iCAMP.Boot.",colnames(treat)[i],".rda"))
write.csv(icboot$summary,file =
paste0(prefix,".iCAMP.BootSummary.",colnames(treat)[i],".csv"),row.names = FALSE)
write.csv(icboot$compare,file =
paste0(prefix,".iCAMP.Compare.",colnames(treat)[i],".csv"),row.names = FALSE)
i=3
treat.use=treat[,i,drop=FALSE]
icamp.result=icres$CbMPDiCBraya
icboot=iCAMP::icamp.boot(icamp.result = icamp.result,treat = treat.use,rand.time =
rand.time,
                         compare = TRUE,silent = FALSE,between.group =
TRUE,ST.estimation = TRUE)
save(icboot,file=paste0(prefix,".iCAMP.Boot.",colnames(treat)[i],".rda"))
write.csv(icboot$summary,file =
paste0(prefix,".iCAMP.BootSummary.",colnames(treat)[i],".csv"),row.names = FALSE)
write.csv(icboot$compare,file =
paste0(prefix,".iCAMP.Compare.",colnames(treat)[i],".csv"),row.names = FALSE)
i=4
treat.use=treat[,i,drop=FALSE]
icamp.result=icres$CbMPDiCBraya
icboot=iCAMP::icamp.boot(icamp.result = icamp.result,treat = treat.use,rand.time =
rand.time,
                         compare = TRUE,silent = FALSE,between.group =
TRUE,ST.estimation = TRUE)
save(icboot,file=paste0(prefix,".iCAMP.Boot.",colnames(treat)[i],".rda"))
write.csv(icboot$summary,file =
paste0(prefix,".iCAMP.BootSummary.",colnames(treat)[i],".csv"),row.names = FALSE)
write.csv(icboot$compare,file =
paste0(prefix,".iCAMP.Compare.",colnames(treat)[i],".csv"),row.names = FALSE)
```

Violin plot

```
library("ggplot2")
data1=read.csv(file.choose(),row.names=1)
p1<-ggplot(data1,aes(x=Factor,y=value,fill=Factor))+geom_violin(alpha=0.7)
```

```
p2<-p1+geom_jitter(alpha=0.3,col="gray",show.legend=FALSE)
mytheme<-theme_bw()+theme(panel.grid.major= element_line(color = "white"),panel.grid.minor =element_line(color= "white"),legend.title = element_blank())
p1+mytheme
p2+mytheme
```

Heatmap

```
library(RColorBrewer)
library(stats)
library(gplots)
library(pheatmap)


x = read.csv(file.choose(),row.names=1)
x = as.matrix(x)
#mat = data.matrix(x)
#pdf("heatmap.pdf",height=60,width=10)



pheatmap(x, cluster_row=FALSE,
         cluster_col=FALSE,
         fontsize=12, fontsize_row=12,
         cellwidth=48,cellheight=32,
         color = colorRampPalette(colors = c("white","gray","skyblue"))(200),
         #color = colorRampPalette(rev(c("#D73027", "#FC8D59", "#FEE090", "#FFFFBF", "#E0F3F8", "#91BFDB", "#4575B4")))(200),
         #col = redgreen(75),
         #col=cm.colors(256))
         #col = colorpanel(10,low = 'white',mid = 'gray',high='blue'),
         #col = redblue(75),
         border_color="black",
         annotation_names_col=FALSE,
         )
? pheatmap
```

FEAST (fast expectation-maximization for microbial source tracking)

```
Packages <- c("Rcpp", "RcppArmadillo", "vegan", "dplyr", "reshape2", "gridExtra", "ggplot2", "ggthemes")
install.packages(Packages)
lapply(Packages, library, character.only = TRUE)
devtools::install_github("cozygene/FEAST")
```

```
library(Rcpp)
library(RcppArmadillo)
library(vegan)
library(dplyr)
library(reshape2)
library(gridExtra)
library(ggplot2)
library(ggthemes)
library(FEAST)


metadata <- Load_metadata(metadata_path = "metadata_example")
otus <- Load_CountMatrix(CountMatrix_path = "otu_example")
FEAST_output <- FEAST(C = otus, metadata = metadata, different_sources_flag = 1, dir_path
= "~/",
                                        outfile="demo")
otus =t(otus)
metadata <- read.csv()

metadata=read.csv(file.choose(),row.names=1)
otus=read.csv(file.choose(),row.names=1)
otus <- t(otus)
common.sample.ids <- intersect(rownames(metadata), rownames(otus))
otus <- otus[common.sample.ids,]
metadata <- metadata[common.sample.ids,]
if(length(common.sample.ids) <= 1) {
   message <- paste(sprintf('Error: there are %d sample ids in common '),
                                  'between the metadata file and data table')
   stop(message)
}
envs <- metadata$Env
train.ix <- which(metadata$SourceSink=='Source')
test.ix <- which(metadata$SourceSink=='Sink')
num_sources <- length(train.ix)
COVERAGE =    min(rowSums(otus[c(train.ix, test.ix),]))
sources <- as.matrix(rarefy(otus[train.ix,], COVERAGE))
sinks <- as.matrix(rarefy(t(as.matrix(otus[test.ix,])), COVERAGE))
FEAST_output<-FEAST(source=sources, sinks = t(sinks), env = envs[train.ix], em_itr =
EM_iterations, COVERAGE = COVERAGE)
```

Random forest modeling

```
library(randomForest)
set.seed( )
```

```
iris.rf = randomForest(Species ~ ., data=iris, importance=TRUE, proximity=TRUE)
varImpPlot(iris.rf)
iris.mds= cmdscale(1 - iris.rf$proximity, eig=TRUE)
op= par(pty="s")
pairs(cbind(iris[,1:4], iris.mds$points), cex=0.6, gap=0,
      col=c("red", "green", "blue")[as.numeric(iris$Species)],
      main="Iris Data: Predictors and MDS of Proximity Based on RandomForest")
par(op)
print(iris.mds$GOF)
ind=sample(2,nrow(iris),replace=TRUE, prob=c(0.67,0.33))
iris.rf = randomForest(Species ~ ., iris[ind==1,], ntree=1000,
                       nPerm=10, mtry=3, proximity=TRUE, importance=TRUE)
print(iris.rf)
iris.pred = predict(iris.rf, iris[ind==2,] )
table(observed=iris[ind==2,"Species"], predicted=iris.pred)
```

Network analysis

```
library(data.table)
library(reshape2)
library(plyr)
library(dplyr)
library(SpiecEasi)
library(ggplot2)
library(igraph)
library(gridExtra)
library(ggpubr)
library(ggsignif)
library(gplots)
library(devtools)
library(stats)
library(parallel)
library(RColorBrewer)
library(Hmisc)
library(viridis)
library(Matrix)
library(stringr)
library(phyloseq)
library(mdmnets)

#���������

otutable <- import_biom(BIOMfilename = "Potutable.biom")
mapping <- import_qiime_sample_data(mapfilename = "pmap.txt")
phylo <- merge_phyloseq(otutable, mapping)
```

```
phylo

prevdf = apply(X = otu_table(phylo),MARGIN = ifelse(taxa_are_rows(phylo), yes=1, no=2), FUN=function(x){sum(x>0)})
prevdf = data.frame(Prevalence = prevdf, TotalAbundance = taxa_sums(phylo), tax_table(phylo))
prevdf <- prevdf[prevdf$Prevalence > 0,]
prev_lineplot <- get_back_counts_for_line_plotf(prevdf, "Rank6")
prevdf
write.csv(prevdf, file = "C:/Users/chairong/Desktop/prevdf.csv")

#get_back_res_meeting_min_occ <- function(phylo, filter_val_percent = 0.4) {
  # phylo = phyloseq object, filter_val_percent = percentage of samples taxa must be present
  silva_otus <- otu_table(phylo)
  newsilva_taxa <- tax_table(phylo)
  silva_filterobj <- filterTaxonMatrix(silva_otus, minocc = 0.7 * length(rownames(sample_data(phylo))), keepSum = TRUE, return.filtered.indices = TRUE)
  silva_otus.f <- silva_filterobj$mat
  print(dim(silva_otus.f))
  silva_taxa.f <- newsilva_taxa[setdiff(1:nrow(newsilva_taxa), silva_filterobj$filtered.indices), ]
  # print(dim(silva_taxa.f))
  dummyTaxonomy <- c("D_0__dummy", "D_1__", "D_2__", "D_3__", "D_4__", "D_5__", "D_6__")
  silva_taxa.f <- rbind(silva_taxa.f, dummyTaxonomy)
  rownames(silva_taxa.f)[nrow(silva_taxa.f)] <- "0"
  rownames(silva_otus.f)[nrow(silva_otus.f)] <- "0"
  silva_updatedotus <- otu_table(silva_otus.f, taxa_are_rows = TRUE)
  silva_updatedtaxa <- tax_table(silva_taxa.f)


  silva_phyloseqobj.final <- phyloseq(silva_updatedotus, silva_updatedtaxa)
  silva_spiec.out <- spiec.easi(silva_phyloseqobj.final, method = "mb", icov.select.params = list(rep.num = 2))
  silva_spiec.graph <- adj2igraph(getRefit(silva_spiec.out), vertex.attr = list(name = taxa_names(silva_phyloseqobj.final)))

  phylo_and_graph <- list(silva_phyloseqobj.final, silva_spiec.graph)

  #return(phylo_and_graph)

  #silva_spiec_plot <- plot_network(silva_spiec.graph, silva_phyloseqobj.final, type='taxa', color="Rank3", label=NULL)
  # return(silva_spiec_plot) }

phylo_for_net <- phylo_and_graph
```

```
colnames(tax_table(phylo_for_net[[1]])) = c("Kingdom", "Phylum", "Class","Order", "Family", "Genus", "Species")
taxa_info = tax_table(phylo_for_net[[1]])
taxa_info <- as.data.frame(taxa_info)
unassignedOTUs_MDM <- c(
  "NA", "uncultured", "uncultured bacterium", "Unknown Phylum",
  "uncultured", "uncultured bacterium", "Unknown Class",
  "Ambiguous_taxa", "Unassigned", "uncultured", "uncultured bacterium", "Unknown Order",
  "uncultured", "uncultured bacterium", "Unknown Family", "uncultured", "uncultured bacterium",
  "Unknown Genus", "uncultured", "uncultured bacterium", "Unknown Species")

taxa_info$Phylum[taxa_info$Phylum %in% unassignedOTUs_MDM] <- "<NA>"
taxa_info$Class[taxa_info$Class %in% unassignedOTUs_MDM] <- "<NA>"
taxa_info$Order[taxa_info$Order %in% unassignedOTUs_MDM] <- "<NA>"
taxa_info$Family[taxa_info$Family %in% unassignedOTUs_MDM] <- "<NA>"
taxa_info$Genus[taxa_info$Genus %in% unassignedOTUs_MDM] <- "<NA>"
taxa_info$Species[taxa_info$Species %in% unassignedOTUs_MDM] <- "<NA>"
taxa_info[] <- lapply(taxa_info, as.character) #convert to character
taxa_info[] <- lapply(taxa_info, function(x) { str_replace_all(x, "uncultured", NA_character_)}) #replace all uncultured to NA


taxa_info <- as.matrix(taxa_info)
taxa2 <- tax_table(taxa_info) #convert to phyloseq format
final_phylo <- merge_phyloseq(otu_table(phylo_for_net[[1]]), taxa2)

colnames(tax_table(final_phylo)) = c("Kingdom", "Phylum", "Class","Order", "Family", "Genus", "Species")
phylo_nets <- get_net_plots_all_ranks(orig_graph = phylo_for_net[[2]], orig_phylo = final_phylo, met_name = "Environment/Data name here")
library(cowplot)
dev.new()
plot_grid(plotlist = phylo_nets)
#final_phylo
#b=tax_table(final_phylo)
#write.csv(b, file = "C:/Users/chairong/Desktop/b.csv")

#met_wo_unk <- function(orig_phylo) {

nodes_list_m <- list()
  met_tax_table <- tax_table(final_phylo)
  print(dim(met_tax_table))
  met_tax_table <- as.data.frame(met_tax_table)
```

```
  met_tax_table[] <- lapply(met_tax_table, as.character)
  met_tax_table1 <- met_tax_table %>% mutate_all(funs(replace(., is.na(.), "Unassigned")))
  rownames(met_tax_table1) <- rownames(met_tax_table)
  print(dim(met_tax_table1))
  for (i in 1:length(colnames(met_tax_table1))) {
    print(i)
    unassignedOTUs_MDM <- c(
      "NA", "uncultured", "uncultured bacterium", "Unknown Phylum",
      "uncultured", "uncultured bacterium", "Unknown Class",
      "Ambiguous_taxa", "Unassigned", "uncultured", "uncultured bacterium", "Unknown
Order",
      "uncultured", "uncultured bacterium", "Unknown Family", "uncultured", "uncultured
bacterium",
      "Unknown Genus", "uncultured", "uncultured bacterium", "Unknown Species")
    node_a <- met_tax_table1[met_tax_table1[, i] %in% unassignedOTUs_MDM, ]
    print(dim(node_a))
    nodes_list_m[[i]] <- node_a
    #print(nodes_list_m[[i]])
    }
  final_otus <- otu_table(final_phylo)
  print(dim(final_otus))
  wo_unk_otus_at_tax_level_l <- list()
  for (t in 1:length(nodes_list_m)) {
    print(t)
    unk_otus_at_tax_level <- final_otus[!rownames(final_otus) %in%
rownames(nodes_list_m[[t]]), ]
    print(dim(unk_otus_at_tax_level))
    wo_unk_otus_at_tax_level_l[[t]] <- unk_otus_at_tax_level }
  names(wo_unk_otus_at_tax_level_l) = colnames(tax_table(final_phylo))
  #return(wo_unk_otus_at_tax_level_l) }

phylo_wo_unk <- wo_unk_otus_at_tax_level_l

phylo_graph_wo_unk <- get_graph_wo_unk(orig_graph = phylo_for_net[[2]], met_wo_unk =
phylo_wo_unk)
phylo_graph = phylo_for_net[[2]]
orig_graph = phylo_for_net[[2]]


phylo_nets_wo_mdm <- lapply(phylo_graph_wo_unk, function(orig_graph){
  new_nets <- get_net_plots_all_ranks(orig_graph, final_phylo, "Predatory mite")
})
library(gridExtra)
dev.new()
```

```
grid.arrange(phylo_nets$Class, phylo_nets_wo_mdm$Class$Class)

phylo_degree_df <- degree_calc_f(orig_graph)

phylo_degree_df_wo_unk <- lapply(phylo_graph_wo_unk, function(orig_graph){df <-
degree_calc_f(orig_graph)})

phylo_comp_net_df <- comp_by_deleting_random_knowns_t_v3(orig_graph,
phylo_graph_wo_unk, final_phylo, phylo_degree_df, phylo_degree_df_wo_unk, iter = 10,
mc.coreval=1)
head(phylo_comp_net_df)

phylo_net_boxplots <- vis_comp_net_meas_boxplots2(phylo_comp_net_df, "Predatory mite",
p_label = "p.signif")
dev.new()
phylo_net_boxplots

phylo_hub_plots <- get_hub_plots_all_ranks(orig_graph, final_phylo, "Predatory mite")

plot_grid(plotlist=phylo_hub_plots)

phylo_bar_and_dens_class_interact <- get_bar_and_dens_class_interactions(orig_graph =
orig_graph,orig_phylo = final_phylo, met_type = "Potworm")
phylo_bar_and_dens_class_interact[[1]]$Unknown
phylo_bar_and_dens_class_interact[[2]]$Unknown
phylo_bar_and_dens_class_interact[[3]]
phylo_bar_and_dens_class_interact[[4]]

hs_sparcc_info <- get_sparcc_info(final_phylo, "Nematode")

hs_sparcc_plot <- plotnet_comp_v2(hs_sparcc_info[[1]], orig_graph, final_phylo, type="taxa",
color="Class", label=NULL) + theme(legend.position="none")
hs_sparcc_plot

c<-hub_score(orig_graph)
write.csv(c, file = "C:/Users/chairong/Desktop/c.csv")
```
